# YANK2 activated by Fyn promotes glioma tumorigenesis via the mTOR-independent p70S6K activation pathway

**DOI:** 10.1038/s41598-024-61157-5

**Published:** 2024-05-07

**Authors:** Yue Shi, Yue Cheng, Wei Wang, Liu Tang, Wensheng Li, Liyuan Zhang, Zheng Yuan, Feng Zhu, Qiuhong Duan

**Affiliations:** 1 Department of Biochemistry and Molecular Biology, School of Basic Medicine, Tongji Medical College, Huazhong University of Science and Technology, Wuhan, 430030 Hubei China; 2 Translational Medicine Center, Huaihe Hospital of Henan University, Kaifeng, 475000 Henan China; 3 Medical and Industry Crossover Research Institute of Medical College, Henan University, Kaifeng, 475000 Henan China; 4 Department of Clinical Laboratory, Zhengzhou Eighth People’s Hospital, Zhengzhou, Henan China

**Keywords:** YANK2, Glioma, Fyn, p70S6K, Tumorigenesis, Cancer, Cell biology, Molecular biology, Oncology

## Abstract

Glioma, particularly glioblastomas (GBM), is incurable brain tumor. The most targeted receptor tyrosine kinase (RTKs) drugs did not bring benefit to GBM patients. The mechanism of glioma growth continues to be explored to find more effective treatment. Here, we reported that Ser/Thr protein kinase YANK2 (yet another kinase 2) is upregulated in glioma tissues and promotes the growth and proliferation of glioma in vitro and in vivo. Further, we confirmed that oncogene Fyn directly activated YANK2 through phosphorylation its Y110, and Fyn-mediated YANK2 phosphorylation at Y110 site promotes glioma growth by increasing its stability. Finally, YANK2 was proved to be a novel upstream kinase of p70S6K and promotes glioma growth by directly phosphorylating p70S6K at T389. Taken together, we found a new mTOR-independent p70S6K activation pathway, Fyn-YANK2-p70S6K, which promotes glioma growth, and YANK2 is a potential oncogene and serves as a novel therapeutic target for glioma.

## Introduction

Glioma, particularly glioblastomas (GBM) is the most common and malignant primary brain tumor in adults. In China, the annual incidence of glioma is 5–8/100,000, and the 5 year mortality rate is second only to pancreatic cancer and lung cancer in systemic tumors (Health Commission-Glioma Diagnosis and Treatment Guidelines 2022 edition). Although brain tumors account for less than 2% of all primary tumors, they account for 7% of cancer life loss before the age of 70^1^. For glioma, the first-line treatment is adjuvant chemotherapy under the premise of maximum surgical resection to control local tumors. However, the prognosis of glioma patients is still poor, the survival period is short, and there is no better therapeutic target^2^. Many aspects of these GBM histologic features remain poorly correlated with key molecular drivers and pathways. For example, the presence or absence of IDH mutations which is the key molecular chromosomal changes in histologic grade III (anaplastic astrocytoma) and GBM (histologic grade IV) tumors cannot be distinguished on pure morphologic grounds in GBM^3^. Therefore, there is an urgent need to explore potential molecular mechanisms and new treatment strategies for glioma.

Yet another kinase 2 (YANK2) is a serine/threonine (Ser/Thr) kinase that belongs to the AGC kinase family, also named STK32B (Ser/Thr protein kinase 32B)^4^. It was found by sequencing human chromosome 4p16.2 and mouse chromosome 5. YANK2 mRNA expression was mainly enhanced in normal kidney and lymphoid tissue and detected in many cancers, such as high expression in adrenocortical cancer and skin cancer (https://www.proteinatlas.org/ENSG00000152953-STK32B/tissue). In 2008, it was first found that the abnormal expression of YANK2 was closely related to Ellis–van Creveld syndrome^5^. It has been reported that YANK2 is associated with various neurological diseases, such as anxiety, essential tremor, primary tremor and Parkinson’s disease^6–8^. Recently, it has been found that YANK2 is associated with the prognosis of oral squamous cell carcinoma and primary hepatocellular carcinoma^9–11^. However, the function and mechanism of YANK2 in glioma remain unclear.

The majority of gliomas exhibited activation of the PI3K/AKT/mTOR pathway and RAS–MAPK signaling pathways^2^. In this study, we found that frequent overexpression of YANK2 in glioma was associated with shorter survival of patients. YANK2 could promote glioma cell proliferation by directly phosphorylating 70 kDa ribosomal protein S6 kinase (p70S6K) at the T389 site. We also found that YANK2 can be activated by Fyn-mediated phosphorylation at Y110, thereby promoting glioma growth.

## Methods

### Human glioma specimens

All human glioma surgical specimens was gifts from Prof. Dongsheng Guo (department of neurosurgery, Tongji Hospital, Tongji Medical College, Huazhong University of Science and Technology), with the informed consents from the patients or their guardians, according to a protocol approved by the Research Ethics Committee of Tongji Hospital, Tongji Medical College, Huazhong University of Science and Technology. At least two nerve pathologists participated in the histopathological diagnosis of glioma specimens based on world Health Organization (WHO) classification. All procedures were performed according to the standard of the Helsinki Declaration and approved by the institutional ethics committees of Tongji Medical College of Huazhong University of Science and Technology.

### Cell culture

The two human glioma cell lines Hs683, U251 were purchased on Shanghai Academy of Biological Sciences. The five human glioma cell lines A172, H4, U118MG, U87MG and U373, the mouse glioma cell line GL261, the rat glioma cell line C6 and the human embryonic renal cell line HEK293T were purchased on ATCC. All cell lines were cultured with DMEM (Gibco, Grand Island, NY) containing 10% fetal bovine serum (FBS, Gibco) at 37 ℃ in a humidified 5% CO_2_ incubator.

### Plasmids and cell lines establishment

YANK2 was purchased on addgene (#23795). pCMV-His-YANK2-Flag, pHAGE-YANK2-Flag, pBind-YANK2, pHAGE-Myc-Fyn-Flag plasmids and pet28a-His-YANK2-His were clone. pCDNA3.1-HA-p70S6K and pCDNA3.1-Flag-mTOR was gift from Prof. Lijun Yao(School of Basic Medicine, Huazhong University of Science and Technology). pCold-His-TF-p70S6K only was gifts from Prof. Yusong Guo (School of Basic Medicine, Huazhong University of Science and Technology). pCMV-His-YANK2-Y110D/Y110F, pet28a-His-YANK2-Y110F-His and pHAGE-Myc-Fyn-Y531F/K299M-Flag were cloned with a Mut Express II fast mutagenesis kit V2 (Vazyme), and the cloning primers are listed in Supplementary Appendix Table S1.

For silencing genes, shRNAs and sgRNA (see Supplementary Appendix Table S1) were designed, cloned and inserted into the pLKO.1 or CRISPR vector to obtain the corresponding plasmids. Lentiviral particles were produced in HEK293T cells transfected with a mixture of plasmid DNA and lentiviral vectors (psPAX2 and pMD2G) with Simple-Fect (Signaling Dawn Biotechnology, Wuhan). The culture medium containing the lentiviruses was collected 48 h after transfection and filtered through a 0.22 μm filter to remove cell debris. The filtered supernatant was added to the cell cultures with polybrene (10 μg/mL) and selected with puromycin (APExBio. Houston, USA) for at least 3–5 days.

For overexpressing genes, transfection methods: GL261 and U87MG cells were transiently transfected with pLVX-LUC-GFP for 48 h and screened with G418 for at least 2 weeks to obtain the GL261/U87MG-Luc cell line; viral infection method: the target DNA sequences were cloned and inserted into the pHAGE vector (such as pHAGE-YANK2-WT/Y110F/Y110D-Flag), and a stable cell line was established by lentiviral packaging (as before).

### Western blotting (WB) and co-immunoprecipitation (IP) assay

For WB, cells were lysed with cell lysis buffer (20 mM Tris pH7.5, 150 mM NaCl, 1% TritonX-100, sodium pyrophosphate, β-glycerophosphate, EDTA, Na_3_VO_4_, leupeptin, Biosharp, BL509A), cell lysates were ultrasonicated at 10% power for 3 min on ice, followed by centrifugation, and protein loading buffer was added to the obtained supernatant and heated at 95 °C for 10 min. For IP, HEK293T cells transiently transfected with the plasmids for 48 h or glioma cells were lysed in co-IP buffer and ultrasonicated at only 5% power, and the whole supernatant was incubated with the corresponding antibody and protein A/G (Santa Cruz) at 4 °C for 12 h on a shaker. Samples were washed with PBS containing 0.1% Triton-100 at least three times, followed by the addition of 2× protein loading buffer at 95 °C for 10 min. The samples were resolved in a 10% SDS–PAGE gel and subjected to immunoblotting. Antibodies for WB are listed in Supplementary Appendix Table S2.

### Bacterial expression

Bacteria (BL21) of pCold-His-TF-p70S6K and pet28a-His-YANK2-His were grown at 37 °C to an absorbance of 0.6–0.8 at A_600nm_, and then 0.5 mM IPTG was added and rotated at 16 °C overnight to an absorbance of 1.2–1.4 at A_600nm_. Bacteria of pet28a-His-YANK2 (WT, Y110F)-His were grown at 37 ℃ to an absorbance of 1.2–1.4 at A_600nm_. The TF-p70S6K protein and His-YANK2-His protein were purified using Ni-NTA agarose (Genscript, L00250), respectively.

### IP-kinase assay and IP mass spectrometry

Active Fyn was prepared by IP according to the following steps: HEK293T cells were transfected with pHAGE-Myc-Fyn-Flag or pCMV-His-YANK2-Flag for 48 h, stimulated with 80 ng/ml EGF for 30 min and harvested for IP of active Fyn or YANK2 using Flag antibody. The inactive human His-YANK2-His or His-TF-p70S6K proteins were purified by Ni-NTA beads from bacteria (as before), and the concentrations were measured using the Bradford method and grayscale scanning. Active Fyn or YANK2 and inactive YANK2 or p70S6K proteins were incubated in 1× kinase buffer in the presence of 100 μM ATP at 37 °C for 2 h. Then, phosphorylated YANK2 or p70S6K was detected by WB using the anti-p-Tyr, anti-p-Ser or anti-p-Thr, and the phosphorylated sites were subsequently analyzed using Thermo’s Q X active plus liquid mass spectrometry system (SpecAlly Life Technology). The enriched proteins and sites in the kinase assay sample are listed in Supplementary Appendix Table S3.

### Immunofluorescent (IF) staining

Cells were seeded in cover glass (Sigma, USA), fixed in 4% paraformaldehyde for 30 min, permeabilized in Triton X-100 for 30 min, and incubated at room temperature with 5% bovine serum albumin (BSA) for 30 min. Then, the samples were washed and incubated with primary antibody in a wet box at 4 °C overnight, washed and incubated with Alexa Fluor 488 (green for p70S6K) or Alexa Fluor 546 (red for YANK2)-conjugated secondary antibody at room temperature in a wet box for 2 h. The nucleus was stained with DAPI. The images were observed and collected under a fluorescence microscope (ZEISS Axioscope5).

### Immunohistochemistry (IHC) and hematoxylin and eosin (HE) staining

Paraffin-embedded mouse or human glioma surgical specimens were baked for 3 h at 60 °C and subjected to microwave treatment in citrate buffer for antigen retrieval. Next, the sections were incubated with 3% H_2_O_2_ for 15 min, blocked with 5% BSA for 1 h and incubated with primary antibodies (Ki67, YANK2, p-p70S6K T389) overnight at 4 °C and secondary antibodies for 30 min. H&E staining was used for histological examination of the brains of tumor-bearing mice. The whole regions of each tumor specimen were observed with an Olympus imaging system microscope and collected by NDP software. view2. The IOD scores of YANK2 in human GBM or mouse GBM models were quantified by software image J under ×400 magnified microscope with an Olympus imaging system microscope in the whole regions of each tumor specimen.

### Cycloheximide (CHX) chase assay

When the cells grew to approximately 80% confluency, the medium was changed to serum-free medium, and the cells were cultured for 12 h. Then, 100 μg/mL CHX (Sigma, C1988) was added to the medium to block new protein synthesis. Cells were harvested at a range of time points for WB to detect the target protein levels. Half-life (t_1/2_) curves were drafted by GraphPad Prism 9 software.

### In vitro growth curve analysis (MTT assay)

Cells (2 × 10^3^/well) were seeded in 96-well plates for 24, 48, 72 and 96 h. Subsequently, 3- (4,5-dimethylthiazol-2-yl)-2,5 diphenyltetrazolium bromide (MTT) (Sigma) was added to each well and incubated for 4 h. Then, 200 μL dimethyl sulfoxide (DMSO) was added, and the absorbance value was measured at a wavelength of A490 nm on an enzyme immunoassay analyzer (Bio-Rad) when the crystal was fully dissolved.

### Plate colony formation assay

Cells (1000 cells/well) were seeded in 12-well plates and cultured in 37 ℃, 5% CO_2_ incubator for 2–3 weeks. The medium was changed every 5 days. After the cells grew to an appropriate density, the cell colonies were washed twice with PBS, fixed with 4% paraformaldehyde for 30 min, stained with 0.1% crystal violet for 30 min, washed once with PBS, and were taken pictures after drying.

### Soft agar colony formation assay

In a 12-well plate, 1.2 ml of agar mix (0.5% Bacto-agar, 10% FBS, 0.33% Eagle’s basic medium, 20 μg/mL gentamycin) was added to each well as bottom agar, 0.8 ml of agar and cell suspension mixture (containing 0.33% Bacto-agar, 3000 cells/well) were used as the top agar and cultured at 37 ℃, 5% CO_2_ for 3–5 weeks. 1 mL medium was replenished every 5 days and colonies were observed under the microscope and photographed.

### Structural analysis and molecular docking of protein

The crystal structure of YANK2 (AF-F1M3G9-F1) and p70S6K (AF-Q9BRS0-F1) were downloaded from Alpha-Fold (https://alphafold.com/) database and imported into Pymol2.3.0 to remove water of crystallization, small molecules, etc. The interaction prediction between YANK2 and p70S6K were docked with HDOCK (http://hdock.phys.hust.edu.cn/). Pymol2.3.0 was used to analyze the interaction patterns of the docking.

### Glioma intracranial mouse models

Four-week-old BALB/c-nu and C57BL/6J mice (Beijing vital river laboratory) from the same batch were randomized into different groups. A total of 2 × 10^5^/10 μl glioma cells (GL261-LUC for C57BL/6J mice and U87MG-LUC for BALB/c-nu mice) were implanted into the right frontal lobe of the intracranial mouse to construct the orthotopic transplanted tumor models. To directly observe tumor growth, 150 mg/kg d-Luciferin (Shanghai Yuanye) was injected intraperitoneally into anesthetized mice, and tumor growth images were acquired by an in vivo imaging system (IVIS) after 5 min. All mice were imaged in second week and weekly measured until the first mouse developed neurological symptoms, then killed when neurological symptoms occurred^12,13^.

The experimental protocol complied with the Hubei Provincial Regulations on the Administration of Laboratory Animals and the regulations of the Laboratory Animal Ethics Committee of HUST, and was approved by the Laboratory Animal Ethics Committee of Tongji Medical College of HUST.

### Statistical analysis

All statistical analyses for the comparison were performed using unpaired t test or one-way analysis of variance (ANOVA). Pearson’s r test was used to examine the correlation of two variables^14^. Bar graphs (GraphPad Prism 9) are presented as the means ± s.d., with statistical significance at *P < 0.05, **P < 0.01, ***P < 0.001. The log-rank test was used to analyze the survival data, with statistical significance at *P < 0.05.

## Results

### High expression of YANK2 correlates with glioma tumorigenicity and prognosis

**Figure 1:**
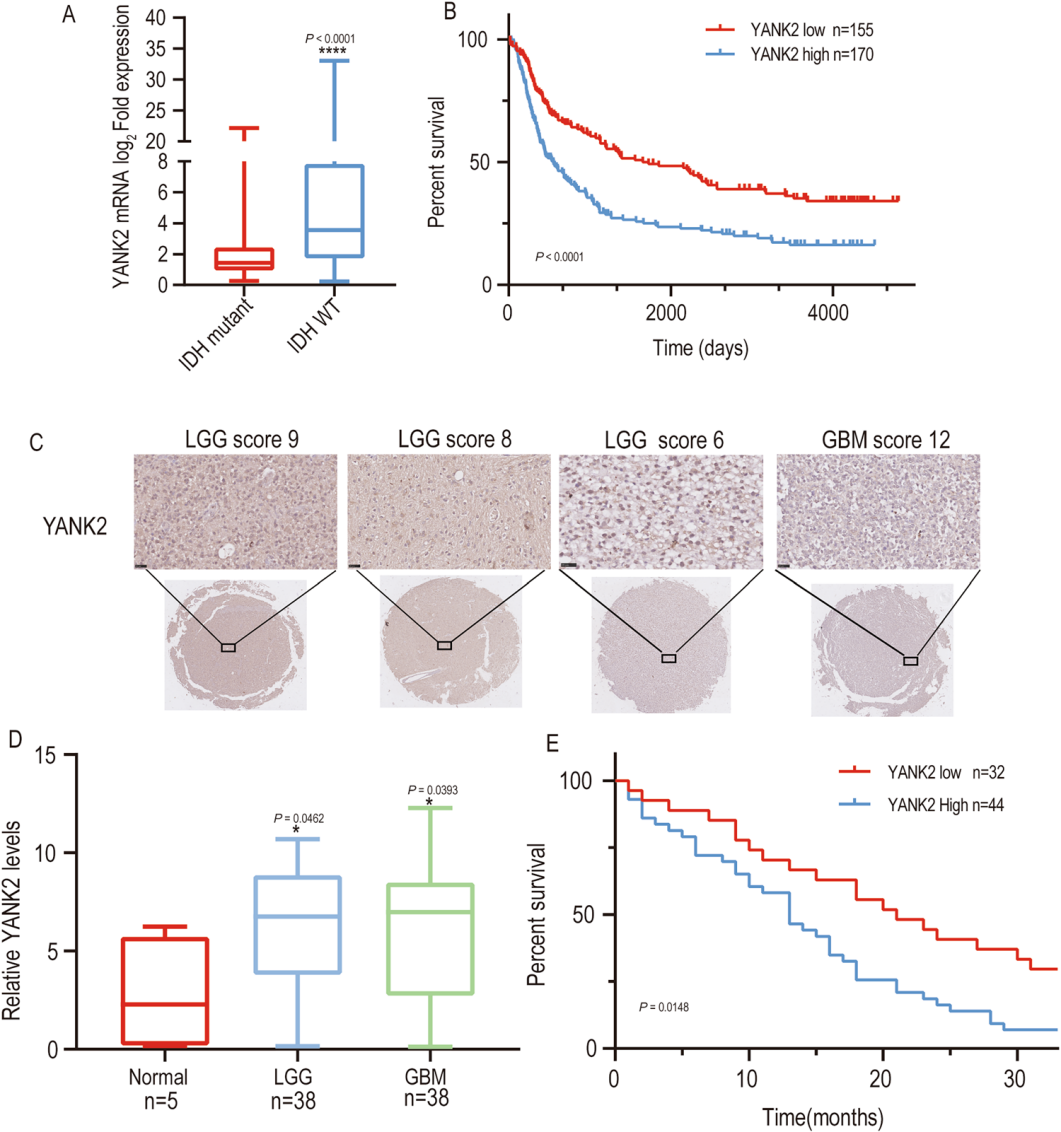
High Expression of YANK2 correlates with tumorigenicity and prognosis of glioma. (**A**) YANK2 mRNA expression in IDH wildtype glioma was higher than that in mutant glioma (data comes from CGGA RNA-sequencing dataset); (**B**) Kaplan–Meier survival analyses for YANK2 mRNA expression in glioma (data comes from CGGA RNA-sequencing dataset); (**C**) immunohistochemistry (IHC) staining with an anti-YANK2 antibody was performed in glioma tissue. Representative images are shown (scale bars, 50 μm); (**D**) IHC Scores of YANK2 of 81 clinical specimens were counted; (**E**) overall survival of glioma patients based on IHC scores of YANK2. Survival curves were built according to the Kaplan–Meier method. Data are expressed as mean and SEM. *P < 0.05, ****P < 0.0001.

Since the role of YANK2 in glioma has not been reported, first, we analyzed The Cancer Genome Atlas (TCGA) and the Chinese Glioma Database (CGGA) and found that YANK2 mRNA levels were not only significantly higher in GBM tissue than in normal brain tissue but also higher in IDH-wide type (IDH-WT) glioma than in IDH-mutant glioma; in addition, these patients with high YANK2 expression also had poor prognosis (Fig. 1A, B and Fig. S1A, B). Then, we investigated the expression of YANK2 in 76 glioma specimens from patients who received treatment at the department of neurosurgery, Wuhan Tongji Hospital at the protein level through IHC and WB. The staining was scored according to staining intensity and the proportion of positively stained tumor cells. The results indicated that YANK2 expression was significantly increased in glioma tissues (n = 76) compared with normal tissues (n = 5) (Fig. 1C and D, Fig. S1C, *P* < 0.05). Kaplan‒Meier survival analysis indicated that patients with higher YANK2 expression (n = 44) tended to have a shorter overall survival (OS) than patients with lower levels of YANK2 (n = 32) (Fig. 1E, *P* < 0.05). Together, we demonstrated that YANK2 expression was clearly increased in glioma tissues and that increased YANK2 expression was a poor prognostic factor for glioma patients.

### Overexpression of YANK2 promotes glioma cells growth and tumorigenicity

**Figure 2:**
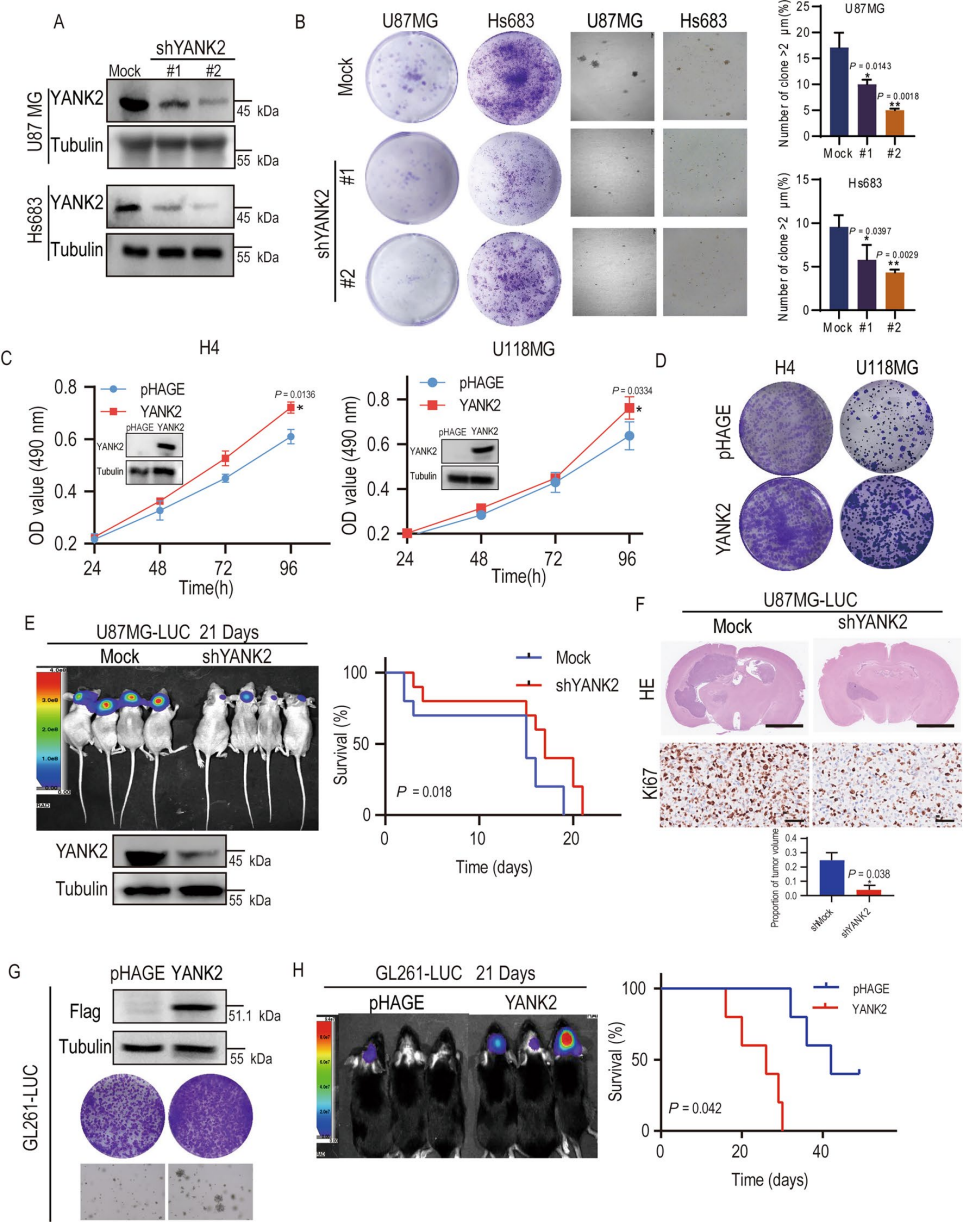
Overexpression of YANK2 promotes cell growth and tumorigenicity of glioma. (**A**) Expression of YANK2 were shown in U87MG-shYANK2 and Hs683-shYANK2 cells by WB (All bands are the result of the same sample, Fig. 2A is the cropped picture, and the original picture is shown in Supplementary raw data Figure 2A); (**B**) Cell clone formation assays were performed in U87MG- or Hs683-shYANK2 cells with representative images (left for plate clone; middle for soft agar) and quantification of spheres with a volume greater than 2 μm in soft agar (right) was shown. The data are presented as the mean ± SEM of three replications. *P < 0.05; **P < 0.01; ***P < 0.001; (**C**) Proliferation of H4- or U118MG-YANK2 stable cells were detected by MTT assay. Insert showed verification of the cell lines identified by WB (All PVDF bands are transferred and tested in the same sample); (**D**) Colony formation assay in H4- or U118MG-YANK2 cells with images acquired after 14 days, n = 3; (**E**) The representative bioluminescence images of BALB/c nude mice with tumors derived from U87MG-LUC-shYANK2 on day 14 (left upper). Left lower showed verification of the cell lines identified by WB. Colored scale bar represents photons/s/cm^2^/steradian. Kaplan–Meier survival curves of mice are shown (right). 2 × 10^5^ cells per mouse, n > 6. (Fig. 2E is the cropped picture, and the original picture is shown in supplemental raw data Figure 2E); (**F**) Hematoxylin and eosin stained (HE) coronal brain sections of tumor xenograft (upper) and the quantified results (lower), IHC of Ki-67 in tumor slices (middle). Scale bars for HE: 2.5 mm. Scale bars for IHC: 100 μm, images of representative tumors were shown. (All PVDF bands are transferred and tested in the same Wb experiment); (**G**) Representative pictures of cell clone formation assays which were performed in GL261-LUC-YANK2 cells and control groups (upper for verification of the cell lines identified by WB; middle for plate clone; lower for soft agar). (All PVDF bands are transferred and tested in the same Wb experiment); (**H**) The representative bioluminescence images of C57 BL/6J mice with tumors derived from GL261-LUC-YANK2 on day 14 (left). Colored scale bar represents photons/s/cm^2^/steradian. Kaplan–Meier survival curves of mice were shown (right). 2 × 10^5^ cells per mouse, n > 6; in (**A**–**H**), Mock and pHAGE were vector control. All bar plot data are means ± SEM. *P < 0.05, **P < 0.01.

To elucidate the effect of YANK2 on glioma growth, we first detected the expression of YANK2 in eight glioma cell lines. The results showed that YANK2 was highly expressed in C6 (Rat), A172, U87MG and Hs683 cells, moderately expressed in U373, U118MG and H4 cells, and poorly expressed in GL261 cells (Mouse) (Fig. S1D). To test whether YANK2 can promote cell growth, YANK2 was knocked down in U87MG and Hs683 cells (Fig. 2A). Next, plate cloning and anchorage-independent growth of shMock and shYANK2 cells were tested, and the results demonstrated that the number of colonies in shYANK2 cells was much less than that in shMock cells (Fig. 2B). Therefore, these results indicated that knockdown of YANK2 in GBM cells inhibited tumorigenesis ex vivo. To verify this idea, YANK2 was further overexpressed in GL261, U118MG and H4 cells. U118MG or H4 stable cell lines that overexpressed pHAGE or pHAGE-YANK2 were generated, and the growth curves of these cells were compared. The results showed that U118MG- or H4-YANK2 cells grew faster than U118MG- or H4-pHAGE cells (Fig. 2C, inner session of left panel indicating YANK2 overexpression). Next, the plate cloning growth of U118MG- or H4-pHAGE or U118MG- or H4-YANK2 was compared, and the results indicated that the number of colonies in U118MG- or H4-YANK2 cell cultures was much greater than that in U118MG- or H4-pHAGE cell cultures (Fig. 2D). These results indicate that YANK2 promotes cell proliferation.

To confirm that YANK2 affects tumor growth in vivo, an orthotopic brain tumor model was utilized. U87MG-LUC cells were employed to stably express shMock or shYANK2 and then intracranially injected into BALB/c nude mice (Fig. 2E). GL261-LUC cells were employed to stably express pHAGE or pHAGE-YANK2 (Fig. 2G, H) and then intracranially injected into C57BL/6J mice. After 14 days, bioluminescence imaging demonstrated that knockdown of YANK2 inhibited U87MG xenograft tumor growth (Fig. 2E left panel) and that overexpression of YANK2 promoted GL261 xenograft tumor growth (Fig. 2H left panel) in vivo. The mice suffering from tumors with shYANK2 had longer neurological symptom-free survival (Fig. 2E, right panel), and the mice suffering from tumors overexpressing YANK2 had shorter neurological symptom-free survival (Fig. 2H, right panel). The HE results showed that tumors in U87MG-shMock-injected mice grew to a much larger size than those in U87MG-shYANK2-injected mice, and the IHC results showed higher expression of Ki67, a marker of cell proliferation activity, in the shMock group (Fig. 2F). Collectively, these results suggested that enhanced YANK2 significantly promotes the proliferation and colony formation of glioma cells in vitro and in vivo.

### YANK2 phosphorylation by Fyn at Y110 promotes glioma growth by increasing its stability

**Figure 3:**
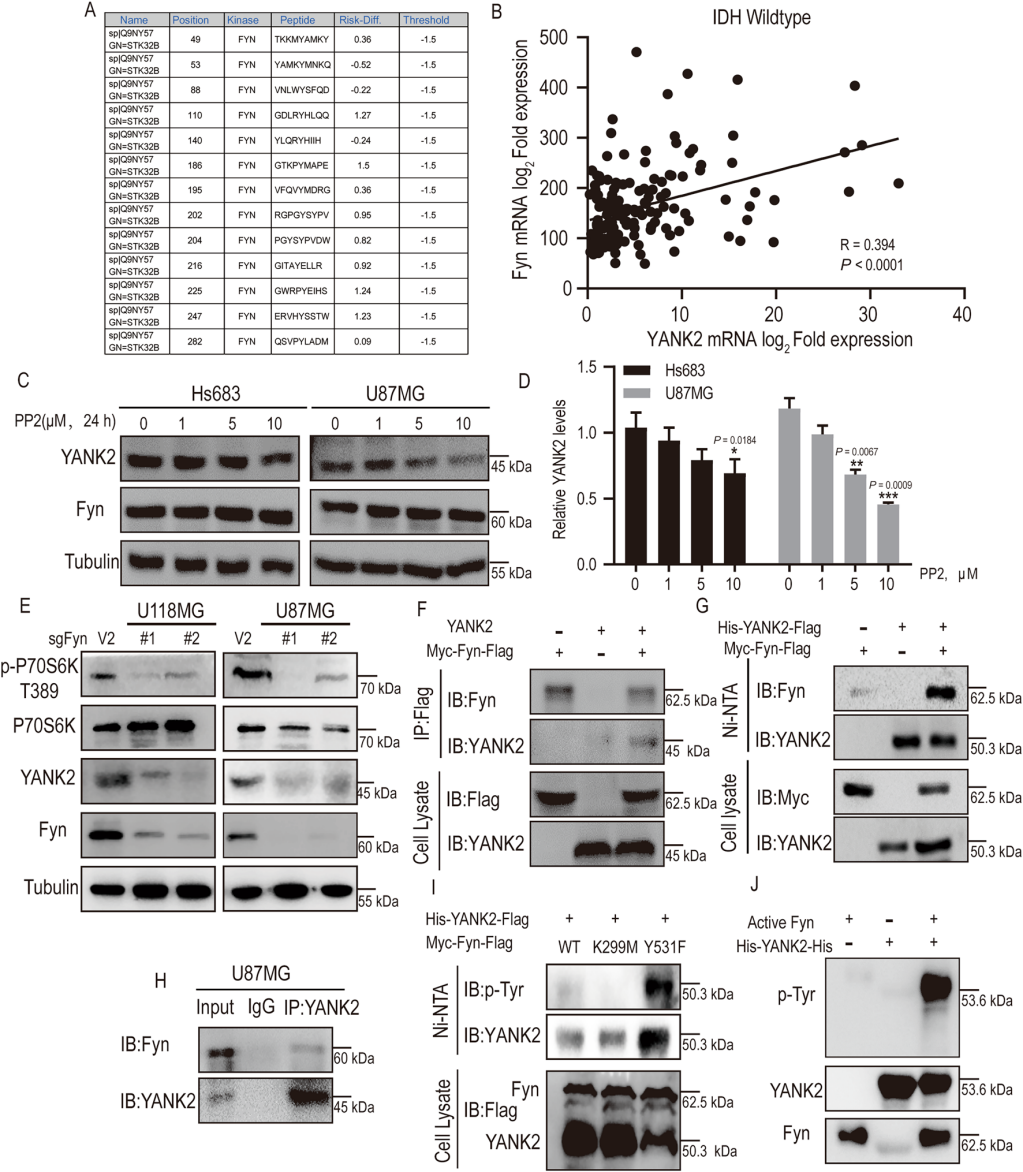
Fyn directly binds with and phosphorylates YANK2 in vitro and ex vivo*.* (**A**) Potential phosphorylated tyrosine sites and upstream kinase of YANK2 were predicted by prediction of PK-specific phosphorylation site website; (**B**) Pearson correlation analysis mRNA levels between Fyn and YANK2 in glioma with IDH-WT (data comes from CGGA RNA-sequencing dataset). Pearson correlation test, R and P values are presented; (**C**,**D**) The level of YANK2 in Hs683 and U87MG threated with different concentration PP2 (Fyn inhibitor) for 24 h was detected by WB (**C**) and the densities of YANK2/Tubulin in Hs683 and U87MG were determined by densitometry (**D**). (All bands are the result of the same sample); (**E**) Expression of YANK2 and Fyn were shown in U118MG- or U87MG-sgFyn cells by WB. (All bands are the result of the same sample); (**F**) pBind-YANK2 or pHAGE-Myc-Fyn-Flag plasmid was transfected or co-transfected into HEK293T cells for 48 h, immunoprecipitated with an anti-Flag, and then probed with anti-YANK2 antibody. (All bands are the result of the same sample); (**G**) pCMV-His-YANK2-Flag or pHAGE-Myc-Fyn-Flag plasmid was transfected or co-transfected into HEK293T for 48 h, pulldown with Ni-NTA beads and then probed with anti-Myc antibody. (All bands are the result of the same sample); (**H**) Endogenous YANK2 was immunoprecipitated from U87MG cells and then probed with anti-Fyn antibody. (All bands are the result of the same sample); (**I**) pCMV-His-YANK2-Flag and pHAGE-Myc-Fyn-Flag (WT, K299M, Y531F) plasmid was co-transfected into HEK293T for 48 h, pulldown with Ni-NTA beads and then probed with anti-p-Tyr antibody. (All bands are the result of the same sample); (**J**) Fyn phosphorylates YANK2 in vitro. Active Fyn was obtained by IP with anti-Flag from HEK293T cells which were transiently transfected with pHAGE-Myc-Fyn-Flag for 48 h and stimulated with EGF (80 ng/ml, 30 min), and then a kinase assay was performed with His-YANK2-His purified from bacteria as substrate, and phosphorylation of YANK2 was detected using anti-p-Tyr antibody by WB. (All bands are the result of the same sample). All bar plot data are means ± SEM. *P < 0.05, **P < 0.01, ***P < 0.001.

**Figure 4:**
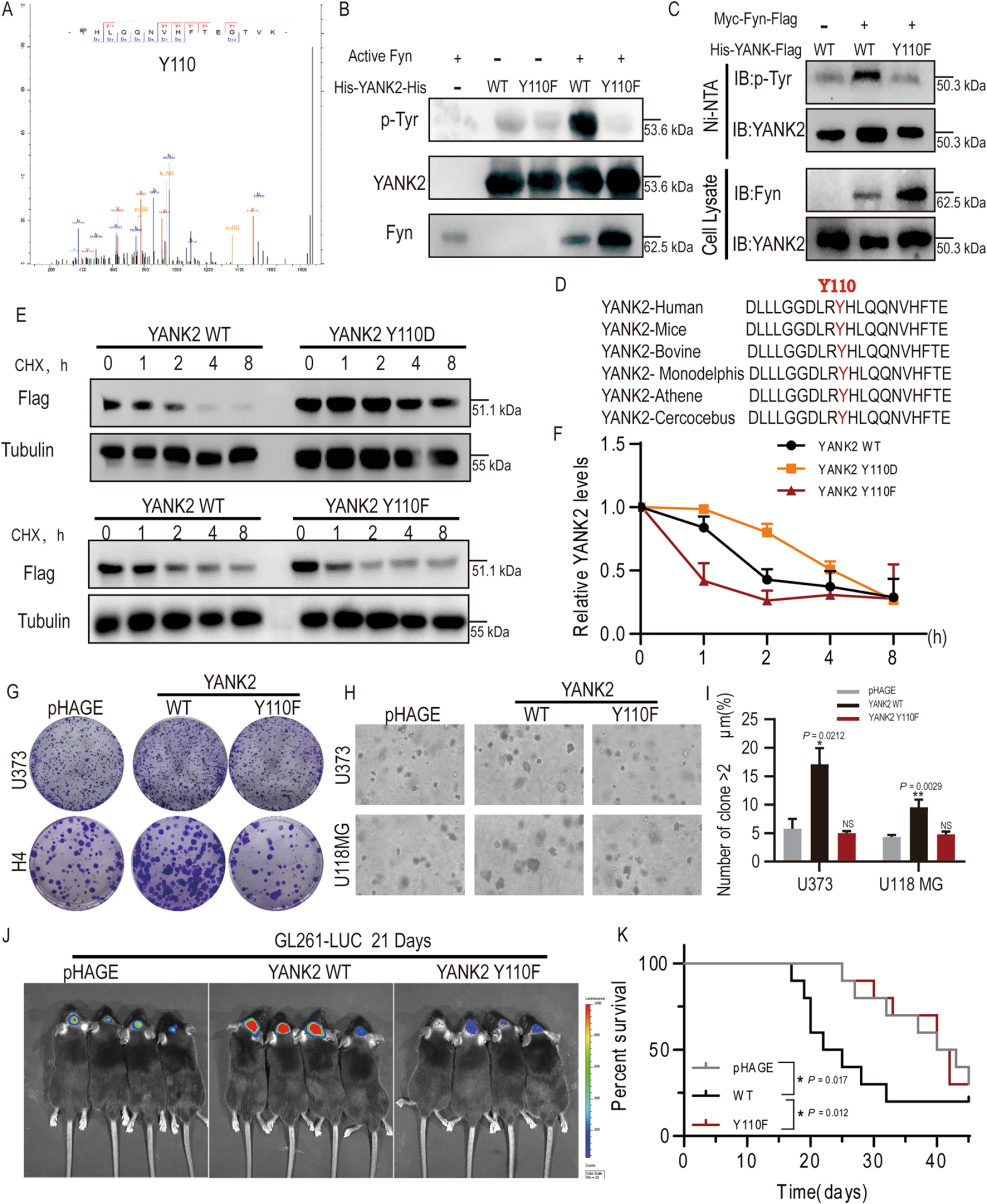
YANK2 phosphorylation by Fyn at Y110 promotes glioma growth by increasing its stability. (**A**) Mass spectrometry (MS) assay was carried out to analyze the phosphorylation modification of YANK2 after an in vitro kinase assay in which Fyn served as an active kinase and His-YANK2-His protein as substrate. The annotated MS/MS spectrum of the phospho-peptide containing the Tyr110 phosphorylated residue of YANK2 was shown. (All bands are the result of the same sample); (**B**) Active Fyn was obtained by IP with anti-Flag from HEK293T cells which were transiently transfected with pHAGE-Myc-Fyn-Flag for 48 h and stimulated with EGF (80 ng/ml, 30 min), and then a kinase assay was performed with His-YANK2-His (WT, Y110F) purified from bacteria as substrate, and phosphorylation of YANK2 was detected using anti-p-Tyr antibody by WB. (All bands are the result of the same sample, Fig. 4B is the cropped picture, and the original picture is shown in supplementary raw data Figure 4B); (**C**) pCMV-His-YANK2-Flag (WT, Y110F) or pHAGE-Myc-Fyn-Flag plasmid was transfected or co-transfected into HEK293T for 48 h, pulldown with Ni-NTA beads and then probed with anti-p-Tyr antibody. (All bands are the result of the same sample, Fig. 4C is the cropped picture, and the original picture is shown in supplemental raw data Figure 4C); (**D**) Amino acid sequence of Y110 residue (red) in YANK2 is highly conserved in different species; (**E**,**F**) The time-dependent stability of YANK2 in H4-YANK2-Flag (WT, Y110D, Y110F) stable cells treated with CHX (100 μg/ml) was analyzed with anti-Flag by WB (**E**) and the densities of flag/tubulin were determined by densitometry (**F**). (All bands are the result of the same sample); (**G**) Plate clone formation assays were performed in U373- or H4-YANK2 (WT, Y110F) stable cells with representative images; (**H**,**I**) Clone formation assays by soft agar were performed in U373- or U118MG-YANK2 (WT, Y110F) stable cells with representative images (**H**) and quantification of spheres with a volume greater than 2 μm in soft agar was shown (**I**). The data are presented as the mean ± SEM of three replications. *P < 0.05; **P < 0.01; (**J**,**K**) The representative bioluminescence images of C57 BL/6J mice with tumors derived from GL261-LUC-YANK2 (WT, Y110F) on day 14 (**J**). Colored scale bar represents photons/s/cm^2^/steradian. Kaplan–Meier survival curves of mice are shown (**K**). 2 × 10^5^ cells per mouse, n > 6; in (**J**,**K**), pHAGE was vector control. All WB analyses are presented as mean ± SEM and representative pictures of three independent experiments. *P < 0.05; **P < 0.01.

The data above indicated that YANK2 could promote glioma growth, and the results were consistent with the positive correlation between YANK2 expression and poor prognosis in human glioma specimens. However, despite the considerable progress in our understanding of the role of YANK2 in glioma, the mechanisms that activate YANK2 during the occurrence and development of glioma have not yet been addressed. To explore the upstream kinase that activates YANK2, we first analyzed its potential phosphorylation sites and upstream kinases by using a website (http://ppsp.biocuckoo.org/). The prediction results indicated that YANK2 has multiple Y sites that may be phosphorylated by the tyrosine protein kinase Fyn (Fig. 3A). Moreover, the CGGC database analysis results showed that the expression of Fyn and YANK2 is positively correlated in glioma (Fig. 3B). Here, we tested the hypothesis that Fyn could directly phosphorylate YANK2 to improve its function. First, we found that the expression of YANK2 in glioma cells treated with PP2 (Fyn inhibitor) or sgFyn was decreased (Fig. 3C–E). Second, the results from IP and pull-down assays confirmed that Fyn could bind with YANK2 (Fig. 3F–H), and the phosphorylation level of YANK2 in Fyn-Y531F (constitutively activated Fyn)-overexpressing 293T cells was significantly increased compared to that in Fyn-WT- or Fyn-K229M (constitutively inactivated Fyn)-overexpressing cells (Fig. 3I). Next, in vitro kinase assay results showed that Fyn directly phosphorylated YANK2 (Fig. 3J). Y110 on YANK2 was identified as a specific phosphorylation site of Fyn by mass spectrometry (MS) (Fig. 4A). Based on the MS results, we then expressed and purified mutant YANK2-Y110F as the substrate of active Fyn for an in vitro kinase assay. Figure 4B shows that the phosphorylation level of YANK2-Y110F was significantly lower than that of YANK2-WT. Moreover, plasmid carrying YANK2-WT or YANK2-Y110F was co-transfected into HEK293T cells with Fyn plasmid. The results showed that the level of p-YANK2 was significantly decreased in the group co-transfected with YANK2-Y110F (Fig. 4C). The above results demonstrated that Fyn is a direct upstream kinase of YANK2 and phosphorylates it at the Y110 site.

Through amino acid sequence comparison analysis, it was found that the sequence containing the Y110 site in YANK2 is highly conserved among biological species, such as human, mice, bovine, monodelphis, athene, cercocebus (Fig. 4D). To verify the biological effects of YANK2 phosphorylation at the Y110 site, we established four cell lines, H4, U373, U118MG or GL261-LUC cells, that stably express YANK2-WT, Y110F (constitutively non-phosphorylated), or Y110D (mimic phosphorylated). We first investigated the t_1/2_ of three types of YANK2 by CHX assay in H4 cells. The results showed that the t_1/2_ of YANK2-Y110D in H4 cells was significantly longer than that of YANK2-WT or Y110F (Fig. 4E, F), which indicated that phosphorylation of YANK2 at the Y110 site could increase its stability. Next, we demonstrated that the cells with YANK2-Y110F grew slower than those with WT by colony formation (Fig. 4G) and soft agar assays (Fig. 4H, I). Subsequently, we validated the function of phosphorylation sites in vivo and found that tumors with YANK2-Y110F exhibited significantly slower growth than those with YANK2-WT (Fig. 4J), and the mice suffering from tumors overexpressing YANK2-WT transplantation had a much worse prognosis (Fig. 4K). These results all indicated that Fyn-mediated YANK2 phosphorylation at the Y110 site promotes glioma growth by increasing its stability.

### YANK2 promotes glioma growth by directly phosphorylating p70S6K at T389

**Figure 5:**
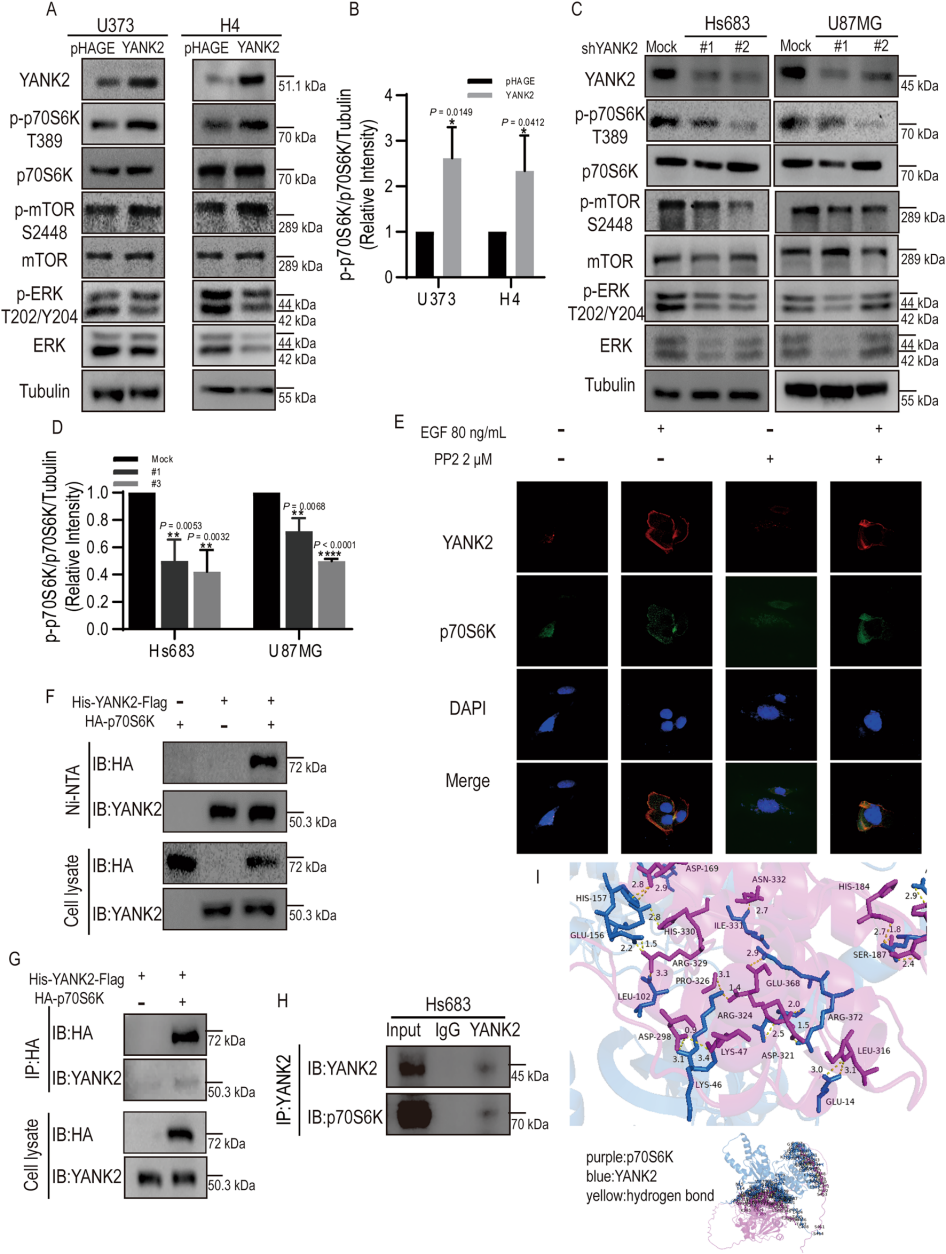
YANK2 can activate the mTOR signaling pathway and directly bind to p70S6K. (**A**,**B**) Key molecules of MAPK and mTOR signaling pathways in U373- or H4-YANK2 stable cells were detected by WB (**A**) and the densities of p-p70S6K/p70S6K/Tubulin were determined by densitometry (**B**). (All bands are the result of the same sample, Fig. 5A is the cropped picture, and the original picture is shown in supplemental raw data Figure 5A); (**C**,**D**) Key molecules of MAPK and mTOR signaling pathways in U87MG- or Hs683-shYANK2 stable cells were detected by WB (**C**) and the densities of p-p70S6K/ p70S6K/Tubulin were determined by densitometry (**D**) (all bands are the result of the same sample, Fig. 5C is the cropped picture, and the original picture is shown in supplemental raw data Figure 5C); (**E**) U118MG cells treated with EGF (80 ng/ml, 30 min) or PP2 (2 μM) were fixed and stained for YANK2 (red), p70S6K (green) and DAPI (blue). Images of representative fluorescence cells are shown (scale bar = 25 μm); (**F**,**G**) pCMV-His-YANK2-Flag or pcDNA3.1-HA-p70S6K plasmid was transfected or co-transfected into HEK293T for 48 h, pulldown with Ni-NTA beads and then probed with Anti-HA antibody (**F**), or immunoprecipitated with an anti-HA, and then probed with anti-YANK2 antibody (**G**) (all bands are the result of the same sample, Fig. 5G are the cropped picture, and the original picture are shown in supplemental raw data Figure 5G); (**H**) Endogenous YANK2 was immunoprecipitated from Hs683 cells and then probed with anti-p70S6K antibody (all bands are the result of the same sample, Fig. 5H are the cropped picture, and the original picture are shown in supplementary Figure 5H); (**I**) Structural model of the protein–protein interaction between YANK2 (blue) and p70S6K (purple). Asn-116, Arg-85, Glu-77, Tyr-63, Gln-75, Thr-54 and Arg-51 of YANK2 form hydrogen bonds with Tyr-90, Asn-226, Phe-232, Trp-214, Tyr-210, Tyr-249 and Glu-251 of p70S6K with hydrogen bond lengths of 3.5 Å, 2.7 Å, 2.6 Å, 2.4 Å, 3.0 Å, 3.1 Å and 2.9 Å, respectively; in (**A**–**D**), pHAGE and Mock were vector control. All WB analyses are presented as mean ± SEM and representative pictures of three independent experiments. *P < 0.05, **P < 0.01, ****P < 0.0001.

Although we confirmed that Fyn mediates YANK2 phosphorylation and promotes glioma growth ex vivo and in vivo, the downstream signaling pathway of YANK2 has not been reported. Next, we wanted to know which signaling pathway was involved in this process. The MAPK and mTOR signaling pathways have been widely reported to be linked with carcinogenesis, with p70S6K, mTOR and ERK being three critical kinases in these two pathways^3^. Therefore, we detected changes in these key molecules in YANK2 or shYANK2 stable cells. The results showed that the expression of p-p70S6K and p-mTOR was increased in H4 or U373 cells stably expressing YANK2, but that of p-ERK did not change (Fig. 5A, B), while the levels of p-p70S6K expression were downregulated in U87MG or Hs683 cells stably expressing shYANK2, and the levels of ERK and p-ERK were downregulated only in U87MG cells stably expressing shYANK2 (Fig. 5C, D). The same results were obtained in H4 or U373 cells stably expressing YANK2-Y110D or Y110F (Fig. S2A). These results suggested that increased YANK2 expression can activate the mTOR signaling pathway. By activated Fyn of EGF, we found that the expression of YANK2 and p70S6K protein increased, co-localization of YANK2 and p70S6K also increased, while the inhibitor of Fyn decreased this colocalization (Fig. 5E). YANK2 interacts with p70S6K but not with mTOR through pulldown or co-IP assays (Fig. 5F–H, Fig. S2B). Molecular docking experiments also demonstrated that there is a physical interaction interface between YANK2 and p70S6K (Fig. 5I). The above results suggested that YANK2, as a protein kinase, most likely directly binds to p70S6K and phosphorylates it to activate p70S6K.

**Figure 6:**
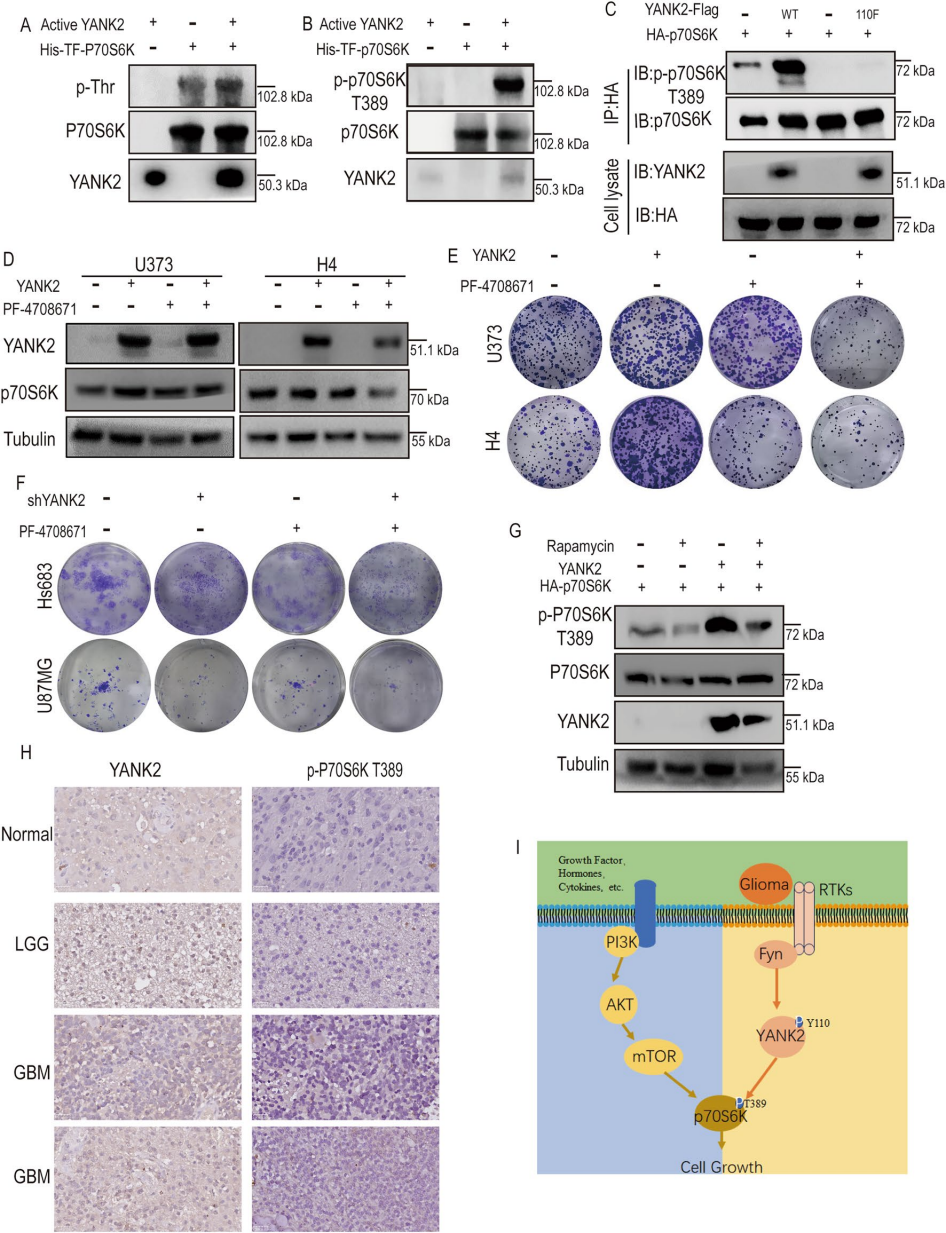
YANK2 promotes glioma growth by directly phosphorylating p70S6K at T389. (**A**,**B**) YANK2 phosphorylates p70S6K in vitro. Active YANK2 was obtained by IP with anti-Flag from HEK293T cells which were transiently transfected with pCMV-His-YANK2-Flag for 48 h and stimulated with EGF (80 ng/ml, 30 min), and then a kinase assay was performed with His-TF-p70S6K purified from bacteria as substrate, and phosphorylation of p70S6K was detected using anti-p-Thr antibody (**A**) or anti-p-p70S6K T389 antibody (**B**) by WB (all bands are the result of the same sample, Fig. 6B are the cropped picture, and the original picture are shown in supplemental raw data Figure 6B); (**C**) YANK2 phosphorylates p70S6K ex vivo*.* pCMV-His-YANK2-Flag or pcDNA3.1-HA-p70S6K plasmid was transfected or co-transfected into HEK293T cells for 48 h, immunoprecipitated with an anti-HA, and then probed with anti-p-p70S6K T389 antibody (all bands are the result of the same sample); (**D**) The level of YANK2 or p70S6K in U373- or H4-YANK2 stable cells threated with 10 μM PF-4708671 (p70S6K inhibitor) for a week was detected by WB (All bands are the result of the same sample); (**E**) Plate clone formation assay in U373- or H4-YANK2 stable cells threated with 10 μM PF-4708671 (p70S6K inhibitor) with representative images; (**F**) Plate clone formation assay in Hs683- or U87MG-shYANK2 stable cells threated with 10 μM PF-4708671 with representative images; (**G**) pCMV-His-YANK2-Flag or pcDNA3.1-HA-p70S6K plasmid was transfected or co-transfected into HEK293T cells, and treated with mTOR inhibitor (rapamycin, 10 nm, 24 h), the expression of p-P70S6K were shown by WB (all bands are the result of the same sample, Fig. 6G are the cropped picture, and the original picture are shown in supplemental raw data Figure 6G); (**H**) Immunohistochemical (IHC) for YANK2 and p-p70S6K T389 expression in three clinical glioma patient specimens (scale bars, 25 μm); (**I**) Schematic diagram showing the mechanism of Fyn-YANK2-p70S6K signaling in glioma cells.

YANK2 has been reported to have Thr/Ser protein kinase activity^15^. To test the hypothesis that YANK2 could directly phosphorylate p70S6K to improve its function, we performed in vitro IP kinase experiments using purified prokaryotically expressed p70S6K as the substrate and IP-YANK2 from cells as the active kinase, and then detected the level of phospho-p70S6K using anti-p-Thr or anti-p-Ser antibodies. The results showed that the phosphorylation of p70S6K at Thr sites was significantly increased compared to that at Ser sites (Fig. 6A and Fig. S2C). We further detected the p-p70S6K T389 level using the classic p-p70S6K T389 antibody, and the results showed that YANK2 could increase the phosphorylation of p70S6K T389 in vitro (Fig. 6B). The level of p-p70S6K T389 was upregulated when YANK2-WT but not YANK2-Y110F was overexpressed in 293T cells (Fig. 6C). These results confirmed that YANK2 is a novel upstream kinase of p70S6K and phosphorylates it at the T389 site. To further confirm that YANK2 plays a role in promoting glioma growth through phosphorylation of p70S6K, H4 or U373 with YANK2, U87MG or Hs683 cells stably expressing shYANK2 were treated with the p70S6K inhibitor PF-4708671, and their growth ability was tested by plate cloning experiments. The results showed that PF-4708671 could significantly inhibit stable YANK2 cell growth and did not reduce YANK2 expression (Fig. 6D, E), but the inhibitory effect of PF-4708671 on stable shYANK2 cells was not obvious (Fig. 6F). Moreover, we further found that phosphorylation of P70S6K also could be detected after treated with the mTOR inhibitor rapamycin in the co-transfection of YANK2 and P70S6K (Fig. 6G), and the p-p70S6K T389 level in YANK2-positive glioma was also increased by IHC assay (Fig. 6H). In summary, the above results indicated that YANK2 promotes glioma growth by directly phosphorylating p70S6K and that there is a novel signaling pathway that is different from mTOR in activating p70S6K in glioma cells (Fig. 6I).

## Discussion

The full name of YANK is yet another novel kinase, which is a kind of Ser/Thr protein kinase, including YANK1, YANK2 and YANK3. It is one of the 24 subtypes of the AGC protein kinase (group of kinases related to PKA, PKG and PKC) superfamily and is in a relatively high position in the evolutionary tree^4^. The YANK family can transfer a γ-phosphate group from ATP to Ser and Thr residues to initiate protein function and catalyze the phosphorylation of substrate proteins^16^. There are few studies on the YANK family, and its biological function is still unclear. Through the analysis of the literature retrieved from the YANK family, it was found that the YANK family was closely related to neurological diseases. Lin MS found that YANK1 was downregulated when they studied the nonmotor disorder mechanism of Huntington’s disease (HD) in mice^17^. In 2021, Carlyle BC found that the change in YANK3 protein levels was significantly correlated with the neuropathology of Alzheimer’s disease (AD)^18^. Many studies have indicated that the YANK2 gene is associated with mental retardation in generalized anxiety disorder, EVC syndrome, essential tremor (ET) and AD^5–8^. In addition, YANK2 was identified as a central gene in the three age stages of fetus, infant and child in the rapid development stage of the prefrontal cortex in Huihui Wang's gene network analysis in 2020, indicating that YANK2 plays an important role in brain development^19^. In Defossez P’s study, YANK2 (GSE124111, GEO Accession viewer (nih.gov)) was found to function in vivo as an activated kinase, and we further explored whether YANK2 plays a role in the glioma process. Our study clearly indicates that YANK2 could dramatically increase the number and size of tumors. Tumors with YANK2 knockdown were largely reduced, demonstrating that YANK2 is essential in glioma. Furthermore, overexpression of YANK2-WT promoted tumor burst in glioma, whereas YANK2-Y110F inhibited this effect. These results indicate that YANK2 is a potential target for glioma tumor therapy.

Many human diseases, including tumors, are caused by gene mutations, abnormal expression and abnormal regulation of protein kinase activity. For example, the amplification and mutation of epidermal growth factor receptor (EGFR) often occur in glioma^20^. The most common mutation is EGFR variant type III (EGFRvIII), which is present in 25%–33% of GBM^21^. It has been proven that the expression of Fyn in glioma patients is closely related to EGFR, and Fyn is a direct effector molecule of carcinogenic EGFR and significantly promotes the invasion of glioma cells with high expression of EGFR^22^. In addition, it has been demonstrated that Fyn kinase is a key player in novel signal transduction that controls neuroblastoma cells development^23^. Andrea Comba demonstrated that Fyn inhibition within glioma cells could improve the efficacy of anti-glioma immunotherapies^24^. Our results also showed that EGF stimulated Fyn to phosphorylate the Y110 site of YANK2, thereby improving its stability. Our results further verified the role of Fyn in promoting the proliferation of glioma cells and identified a new target of Fyn. In our study, we confirmed the pro-cancer role of the FYN/YANK2 axis in glioma progression, and inhibition of YANK2 could significantly delay the progression of glioma.

The p70S6K, which is activated by mTOR, is a Ser/Thr kinase^15^. Activated p70S6K promoted protein synthesis through a variety of mechanisms, including increased translation initiation through the phosphorylation of eIF4B and PDCD4^25^. Deregulation of p70S6K played an important role in the development of many cancers, such as glioma^26^. Targeting of mTOR and p70S6K signaling in cancers, including glioma, has met with limited success. Our results suggested that YANK2, which is independent of mTOR, directly phosphorylates the T389 site of p70S6K to increase the growth of glioma. Since the known phosphorylation site of p70S6K also has many Thr sites, such as T229 and T404^25,27–29^, we will use other commercial antibodies to prove whether YANK2 can phosphorylate other phosphorylation sites of p70S6K. Interestingly, we also observed an increase in mTOR phosphorylation in cells overexpressing YANK2, but no interaction between YANK2 and mTOR was detected. It has been reported that p70S6K can feedback and regulate the activity of mTOR^30^. Therefore, we speculated that YANK2 indirectly caused changes in mTOR by affecting p70S6K. Our study found that glioma cells proliferation was affected by blockade of YANK2/p70S6K. The p70S6K kinase inhibitor PF4708671 inhibited YANK2-high-expression glioma cells proliferation, which is consistent with results from previous studies^31^. These results suggested that YANK2-p70S6K can work well as a target for glioma therapeutic medicine.

## Conclusion

Our data collectively describe the Fyn-YANK2-p70S6K signaling axis, which regulates cell growth activity, tumorigenicity and response to EGF in glioma. The phosphorylation of YANK2 in glioma is regulated by the nonreceptor tyrosine Fyn, and its half-life is significantly enhanced when it is activated by phosphorylation. EGF-induced YANK2 phosphorylates p70S6K at T389, activates p70S6K to promote cell proliferation, enhances the tumorigenicity of glioma cells, and significantly reduces the survival time of glioma patients. Pharmacological inhibition of p70S6K using PF-4708671 can significantly reduce the effect of YANK2 on promoting proliferation and reducing the tumorigenicity of glioma cells, which is a new potential target for the treatment of glioma.

### Supplementary Information

Supplementary Figures.

Supplementary Information.

## Data Availability

The data supporting the findings of this study are available from the corresponding author upon reasonable request.
