# Supplementary material for: YANK2 activated by Fyn promotes glioma tumorigenesis via the mTOR-independent p70S6K activation pathway
Source: Sci Rep. 2024 May 7;14:10507. doi: 10.1038/s41598-024-61157-5 (PMC11076283; doi:10.1038/s41598-024-61157-5)

According to the publication requirements of scientific reports for electrophoretic gels and imprints: the figure legend must state that thesamples derive from the same experiment and that gels/blots were processed in parallel.
To ensure that our bands were performed in the same wb experiment, we considered some proteins with large molecular weight differences, such as mTOR protein with a molecular weight of 289 kda and tubulin with a molecular weight of 55 kda, we transferred the samples to the same membrane, cut open and incubate the antibodies separately; For some small membranes, we cut them before incubating the primary antibody. Some molecules, such as P70S6K (70 kDa), Fyn (60 kDa), YANK2 (45 kDa), and Tubulin (55 kDa), were tested with multiple loads of the same batch of samples due to their close molecular weight. For important IP and in vitro kinase experiments, they are all on the same PVDF membrane. Among them, the image that has been cropped too close, I have indicated in the legend and explained it in the original pictureWe hope that the editors will understand and support our research. Thank you.

Same sample

Figure 2A.

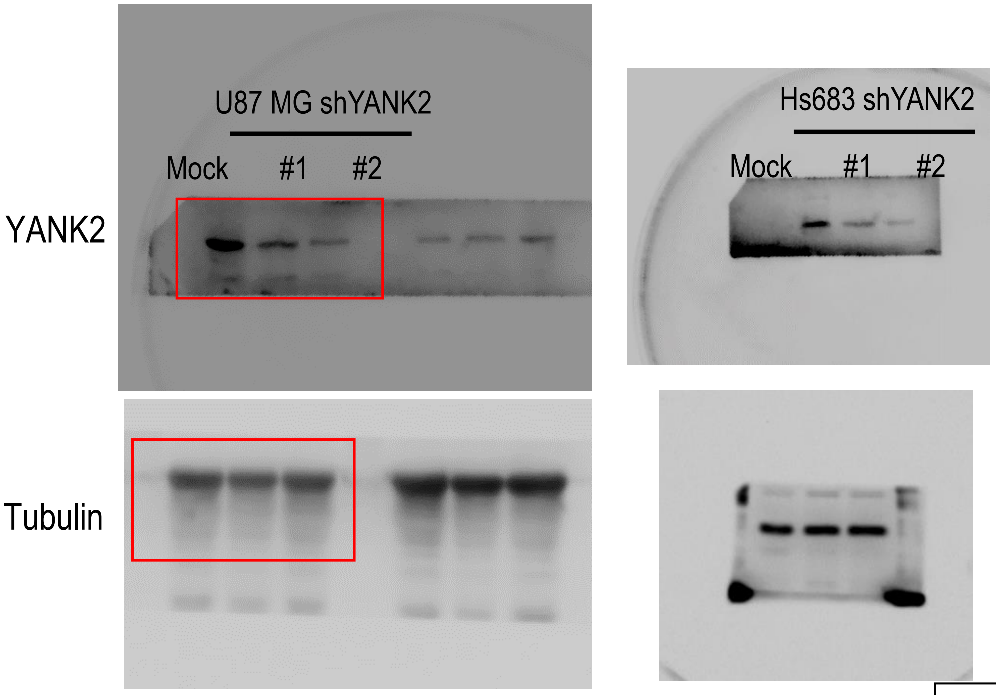

Figure 2E.

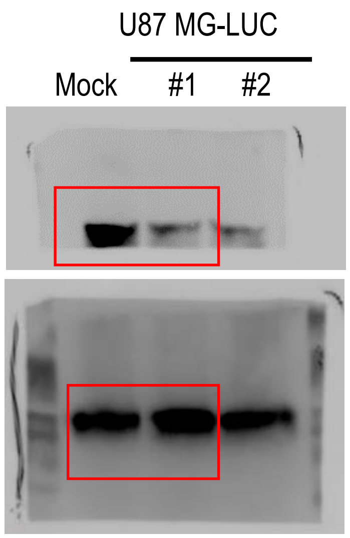

The knockdown effect of silencing cells with two different silencing sequences was detected, and the knockdown cell line of the first sequence was selected for subsequent animal experiments.

Figure 2C.

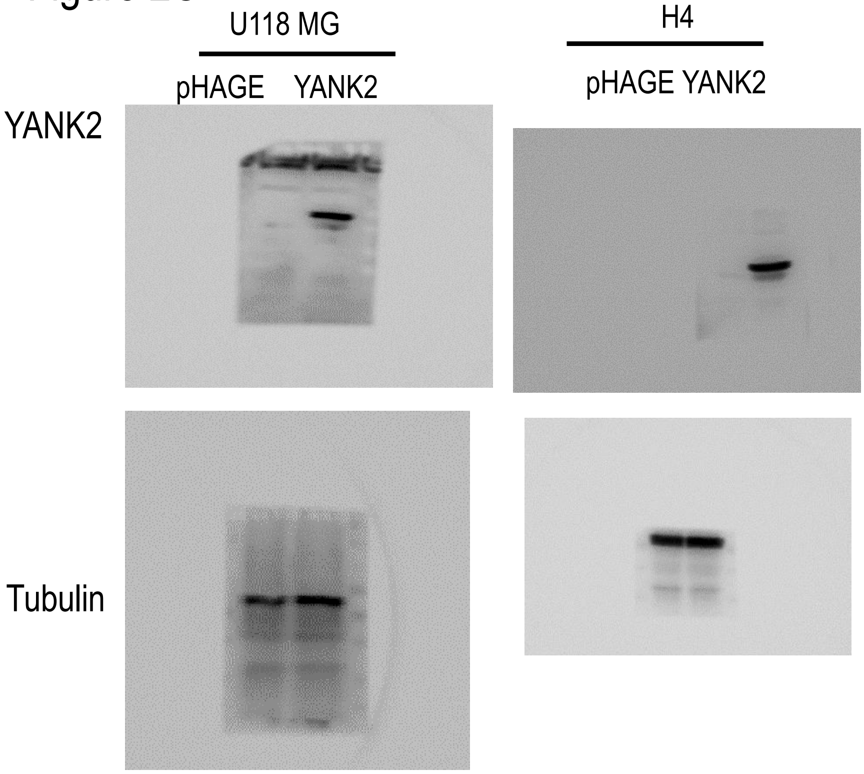

Figure 2G.

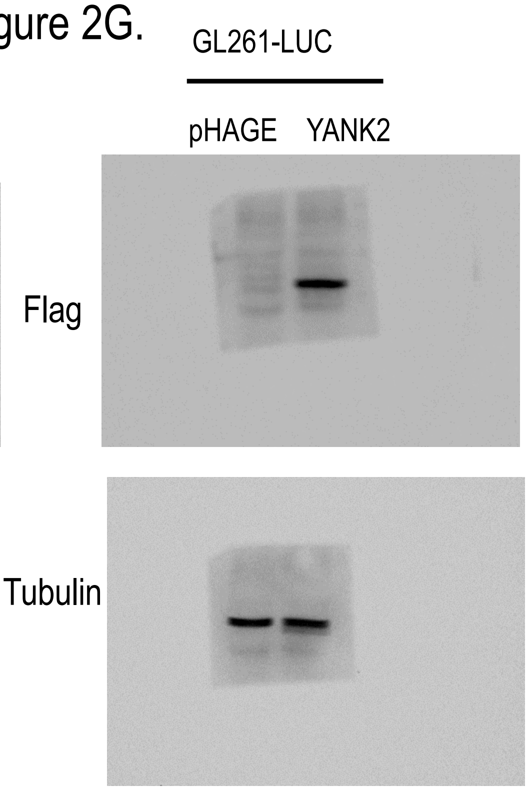

Figure 3C.

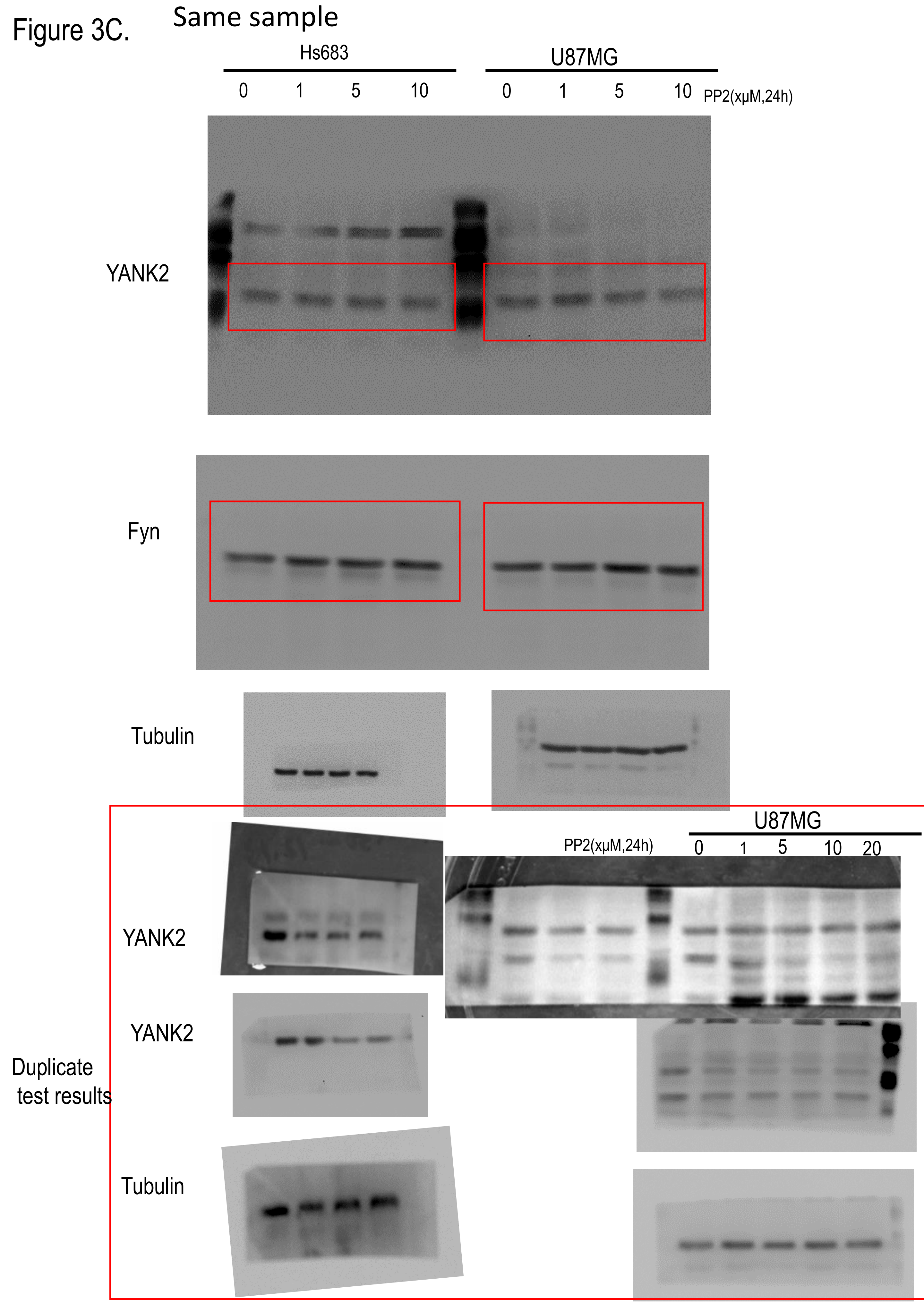
Same sample
Hs683
U87MG
0
1
5
10
0
1
5
10
PP2(xμM,24h)
YANK2
Fyn
Tubulin
Duplicate test results
YANK2
YANK2
Tubulin
U87MG
PP2(xμM,24h)
0
1
5
10
20

Figure3E.

Same sample

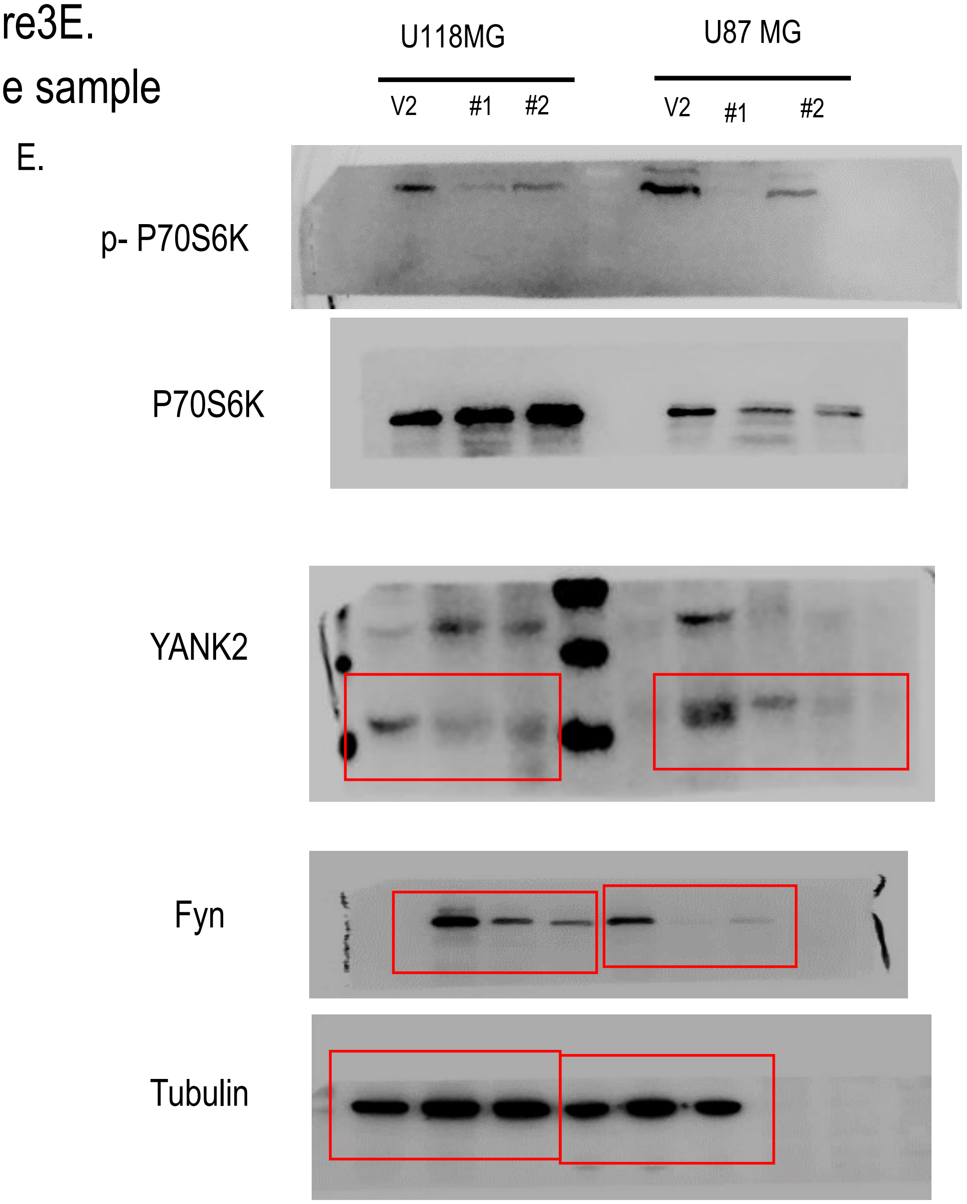

Figure3F.

Same sample

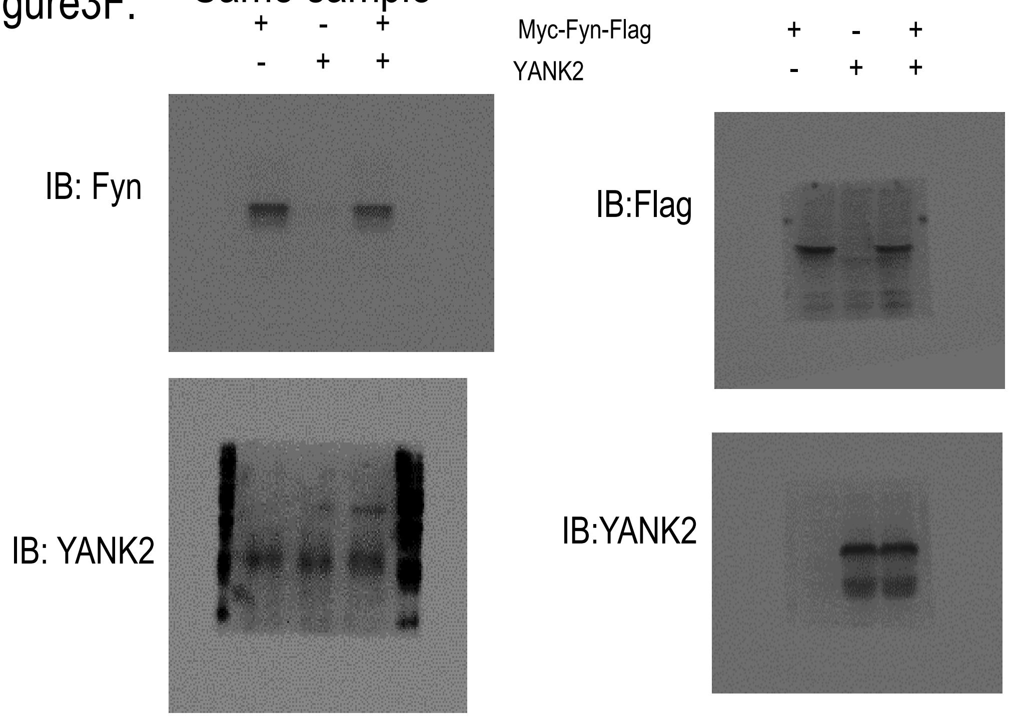

Figure3G. Same PVDF membrane

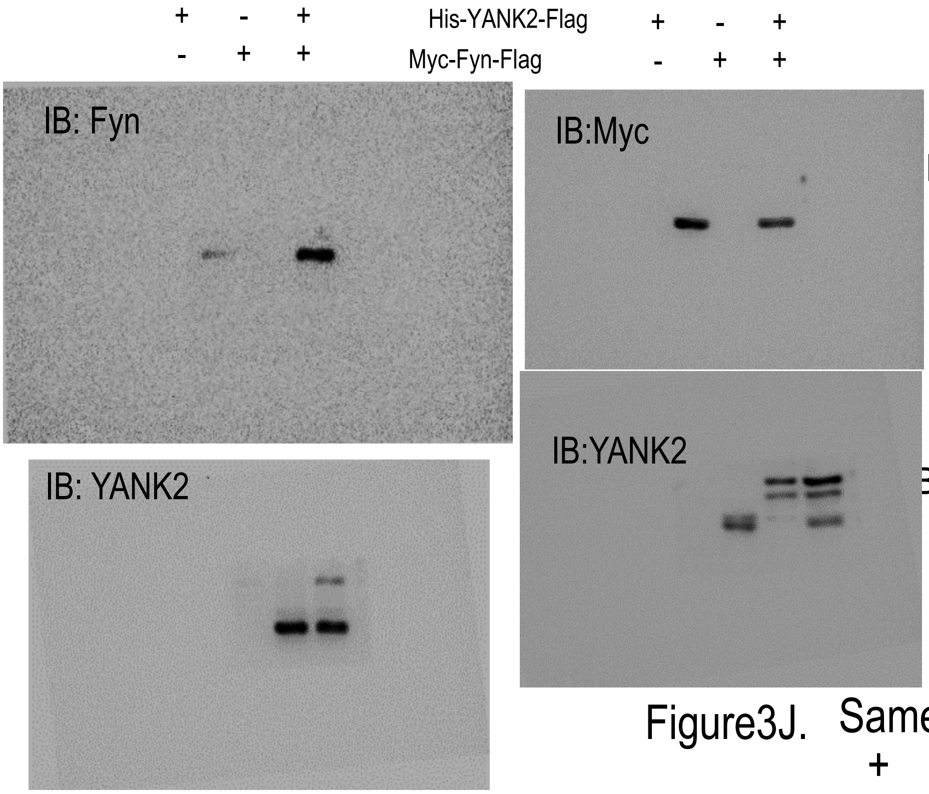

Same PVDF membrane
Figure3H.

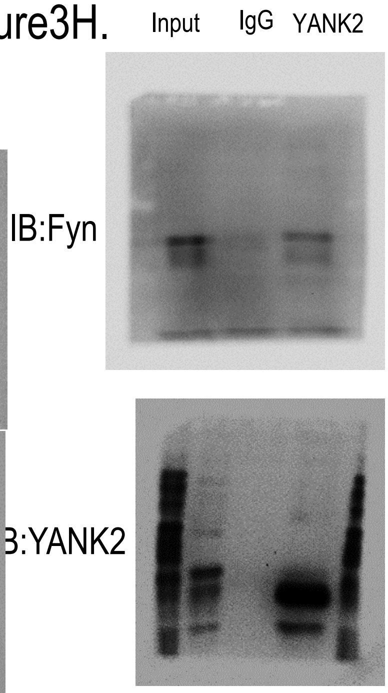

Figure3J. Same PVDF membrane

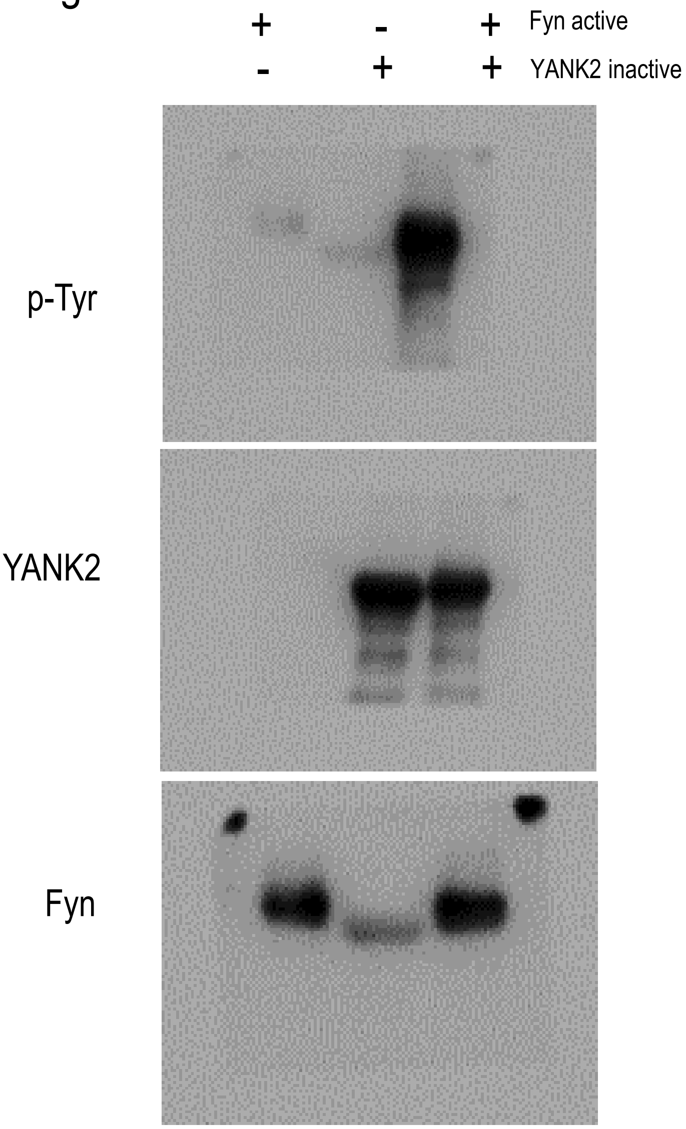

Figure3I. Same PVDF membrane

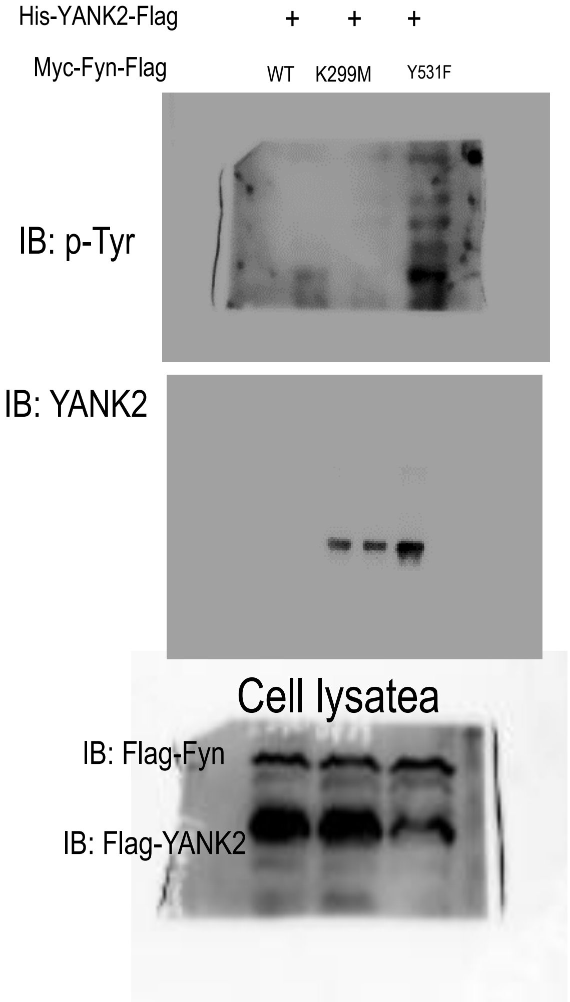

Figure4B.

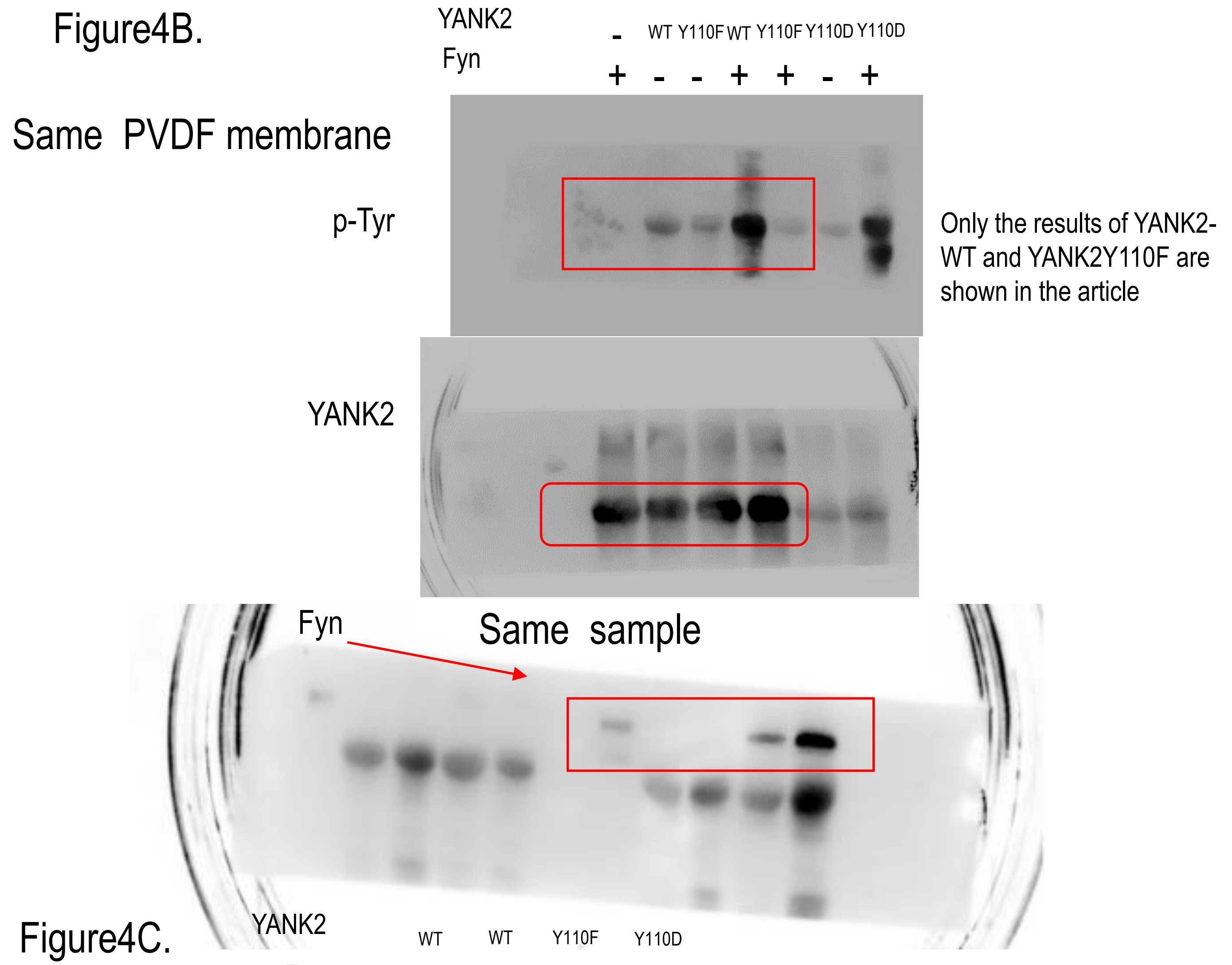

Figure4C.

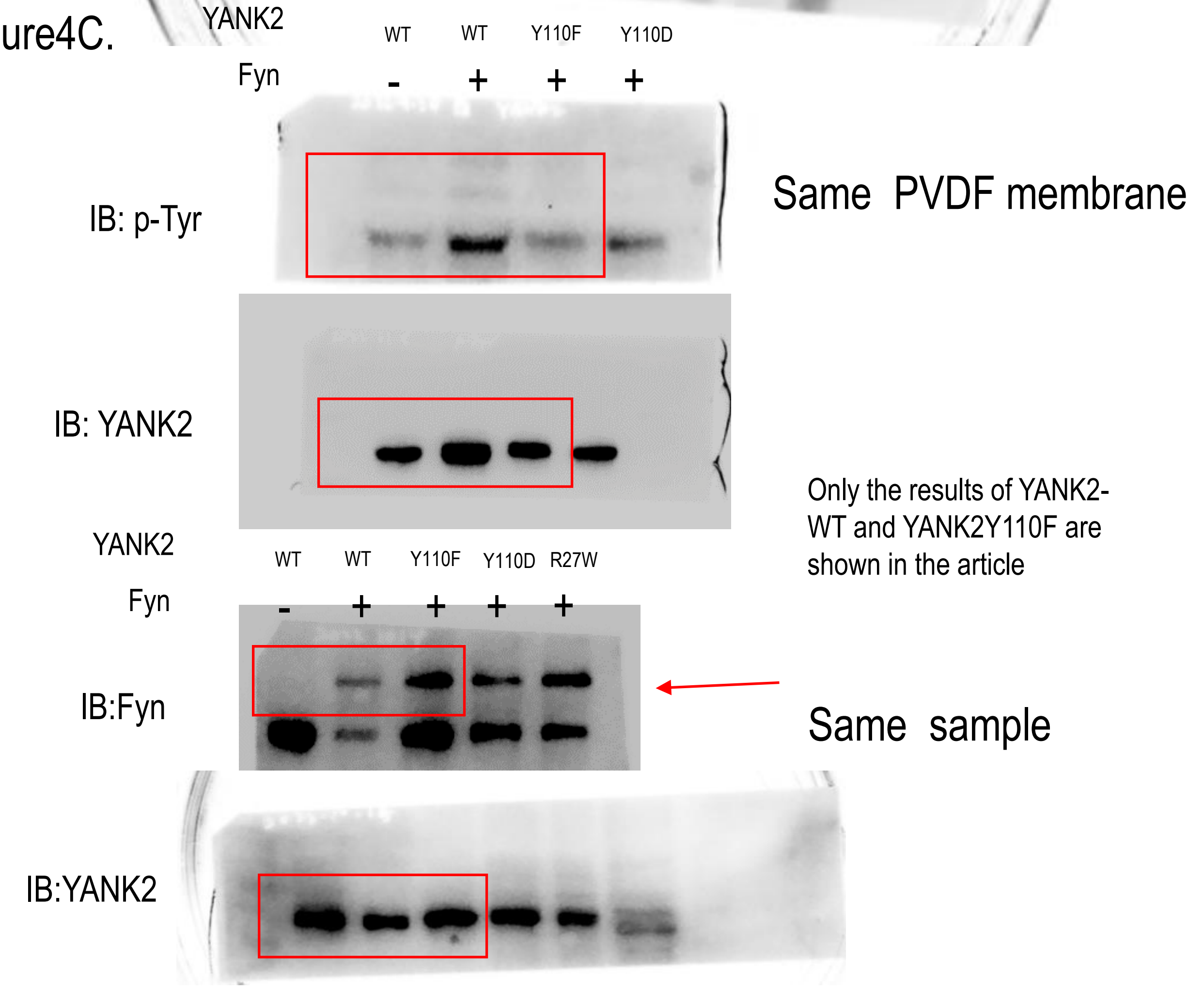

Figure4E.

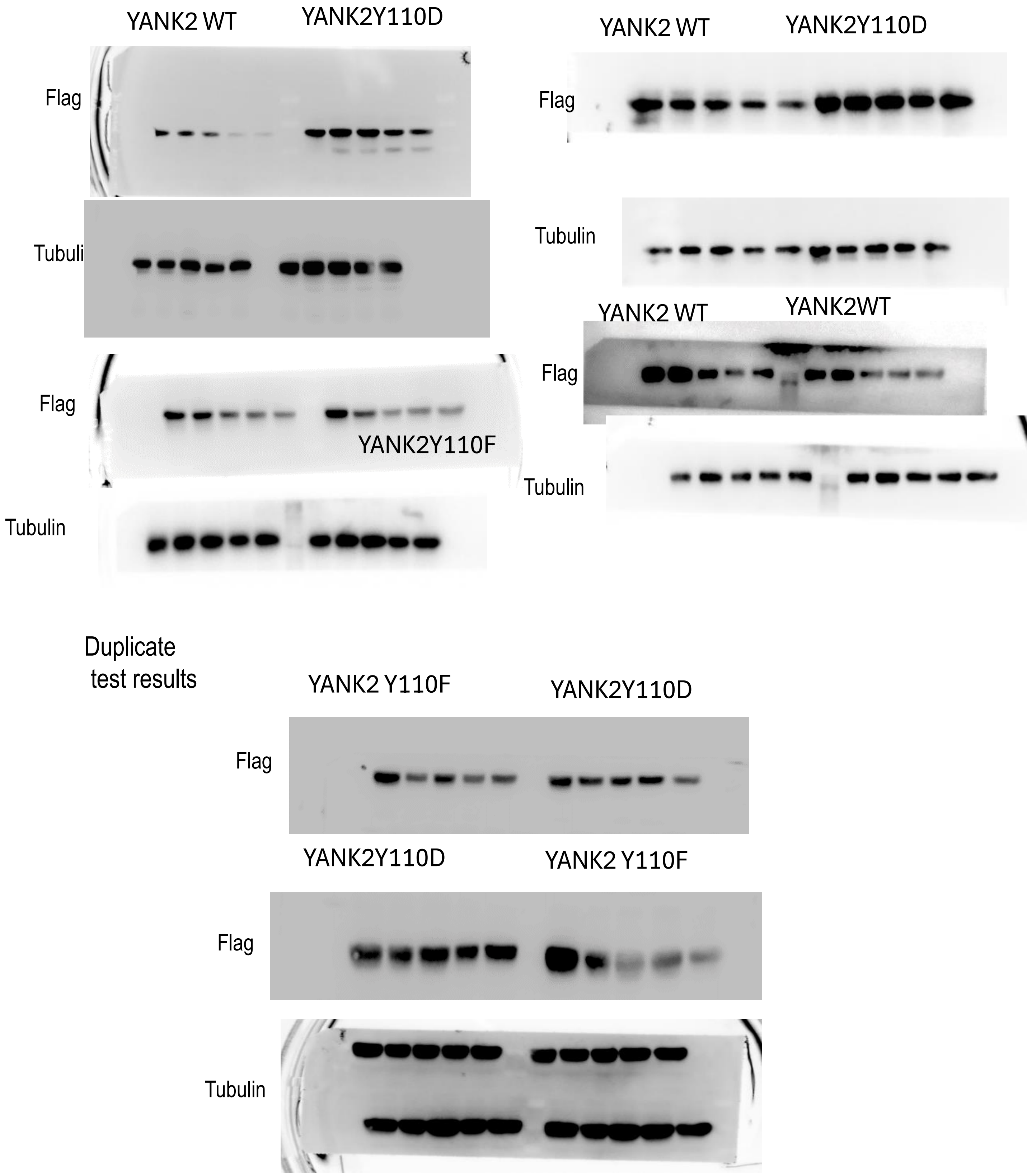
YANK2 WT
YANK2Y110D
Flag
Tubuli
Flag
YANK2Y110F
Tubulin
YANK2 WT
YANK2Y110D
Flag
Tubulin
YANK2 WT
YANK2WT
Flag
Tubulin
Duplicate
test results
YANK2 Y110F
YANK2Y110D
Flag
YANK2Y110D
YANK2 Y110F
Flag
Tubulin

Figure5A.

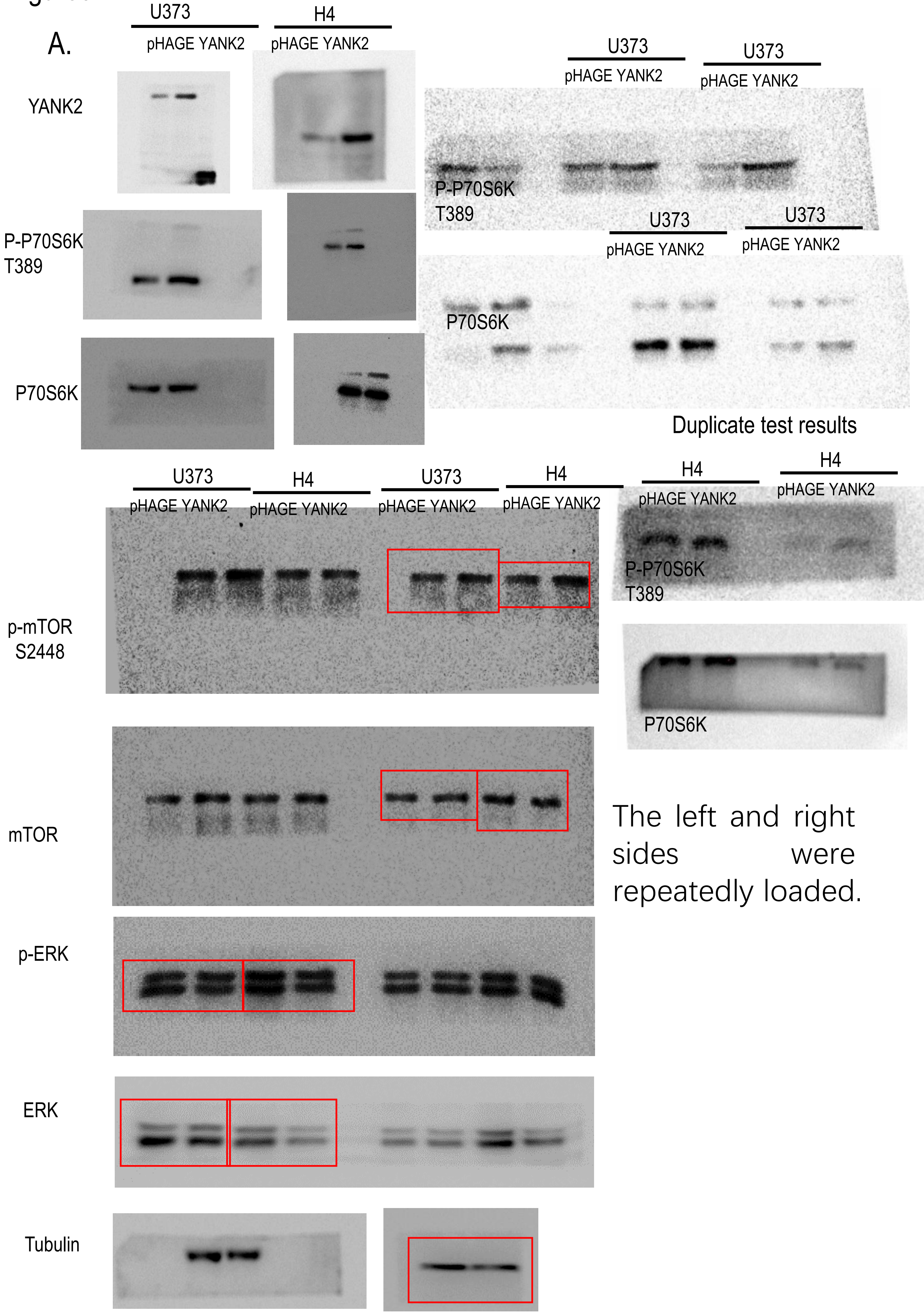

A.
U373
H4
pHAGE YANK2
YANK2
P-P70S6K
T389
P70S6K
Duplicate test results
p-mTOR
S2448
mTOR
p-ERK
ERK
Tubulin
The left and right sides were repeatedly loaded.

Figure5C.

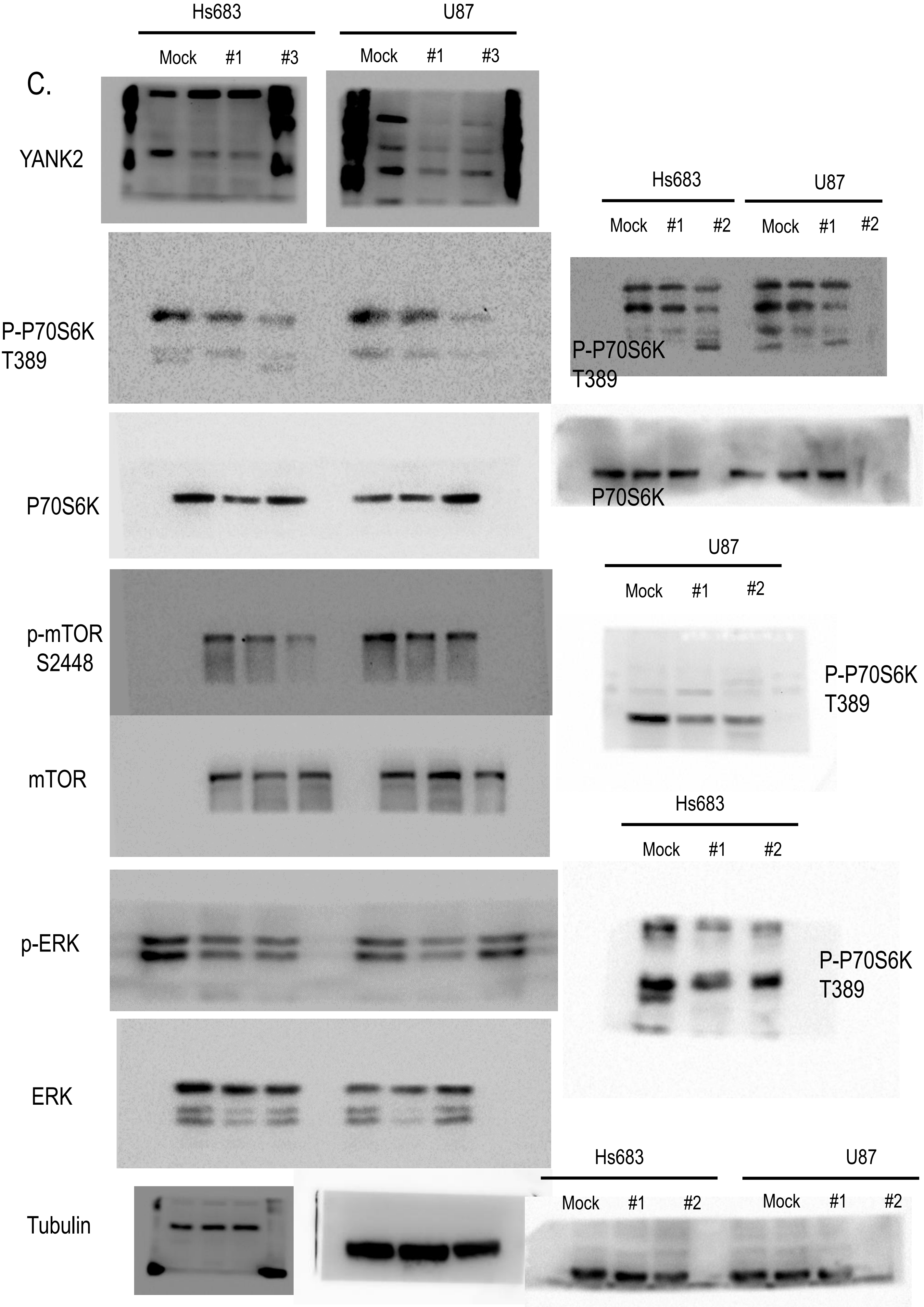
Hs683
U87
Mock
#1
#3
Mock
#1
#3
C.
YANK2
P-P70S6K
T389
P70S6K
p-mTOR
S2448
mTOR
p-ERK
ERK
Tubulin
Hs683
U87
Mock
#1
#2
Mock
#1
#2
P-P70S6K
T389
P70S6K
U87
Mock
#1
#2
P-P70S6K
T389
Hs683
Mock
#1
#2
P-P70S6K
T389
Hs683
U87
Mock
#1
#2
Mock
#1
#2

Figure 5 F.

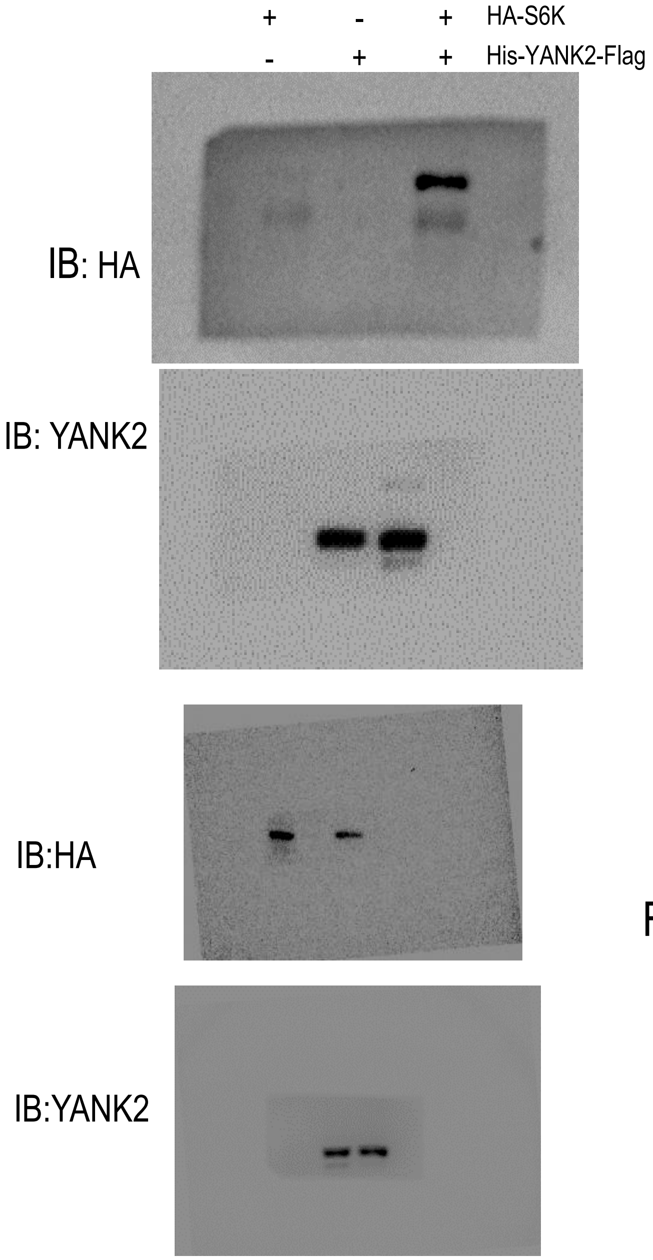

Figure 5 G.

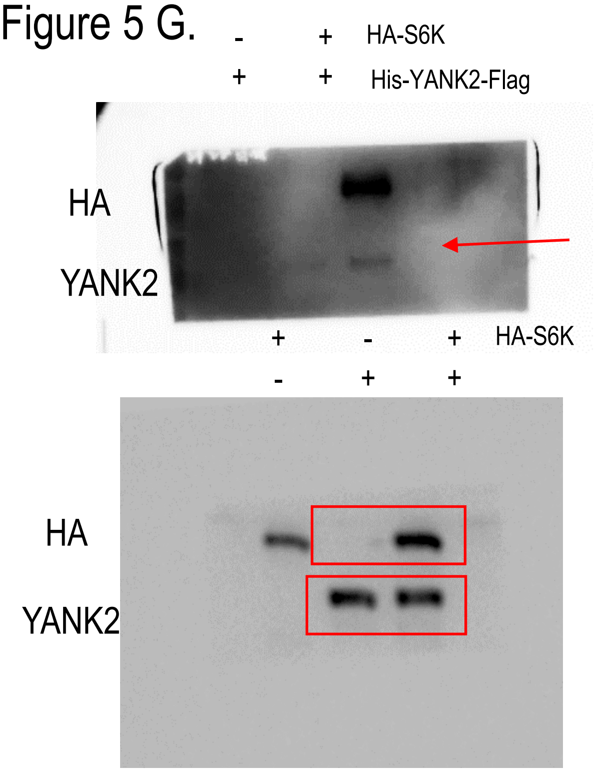

Figure 5H. Same PVDF membrane

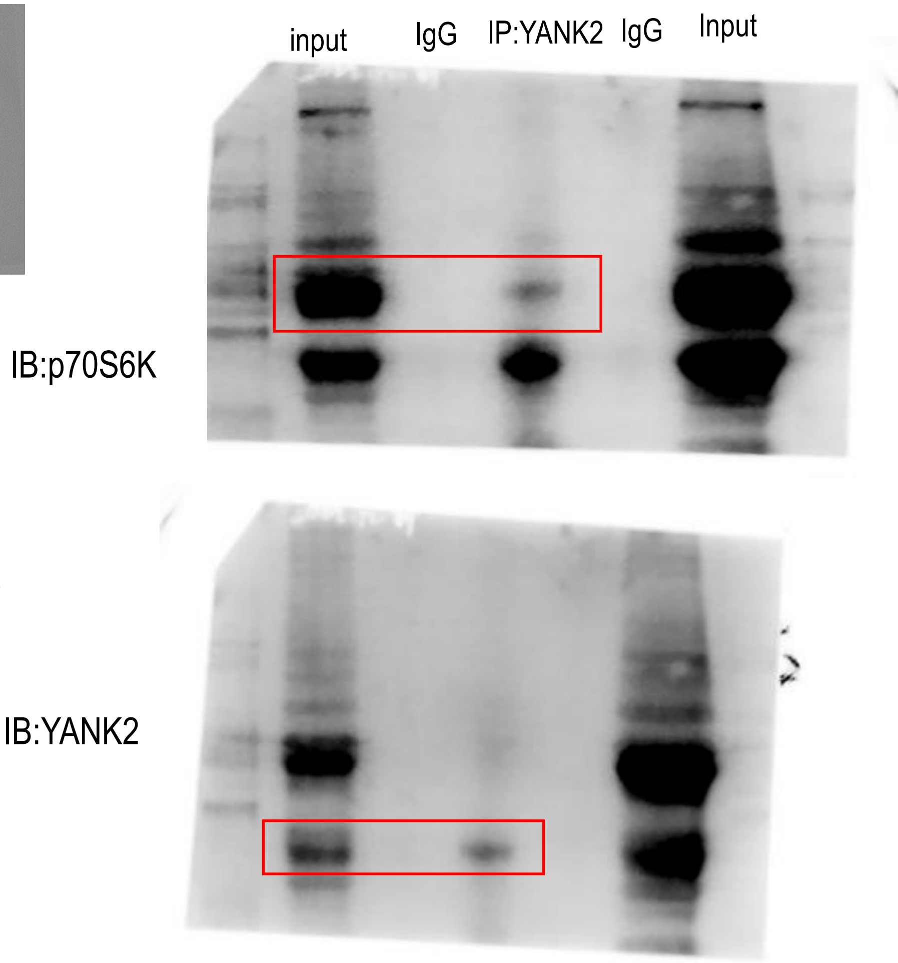

The input loading volume on the left and right sides is inconsistent..

Figure6 A. Same PVDF membrane

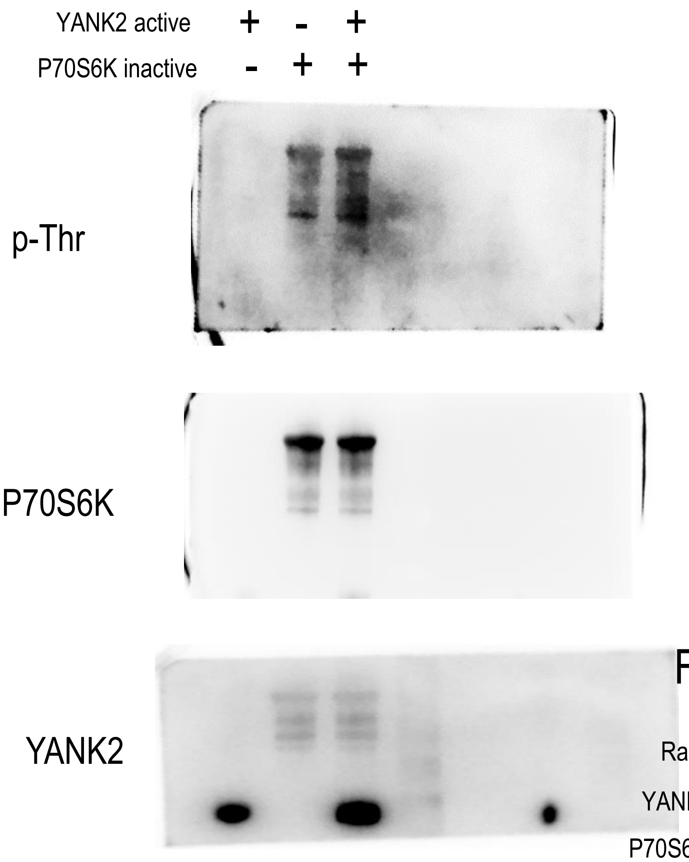

Figure6 B. Same PVDF membrane

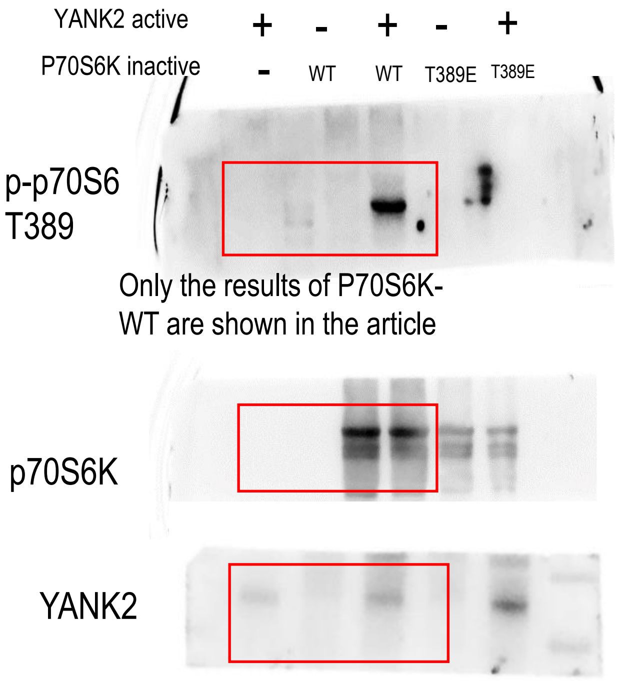

Figure 6C. Same PVDF membrane

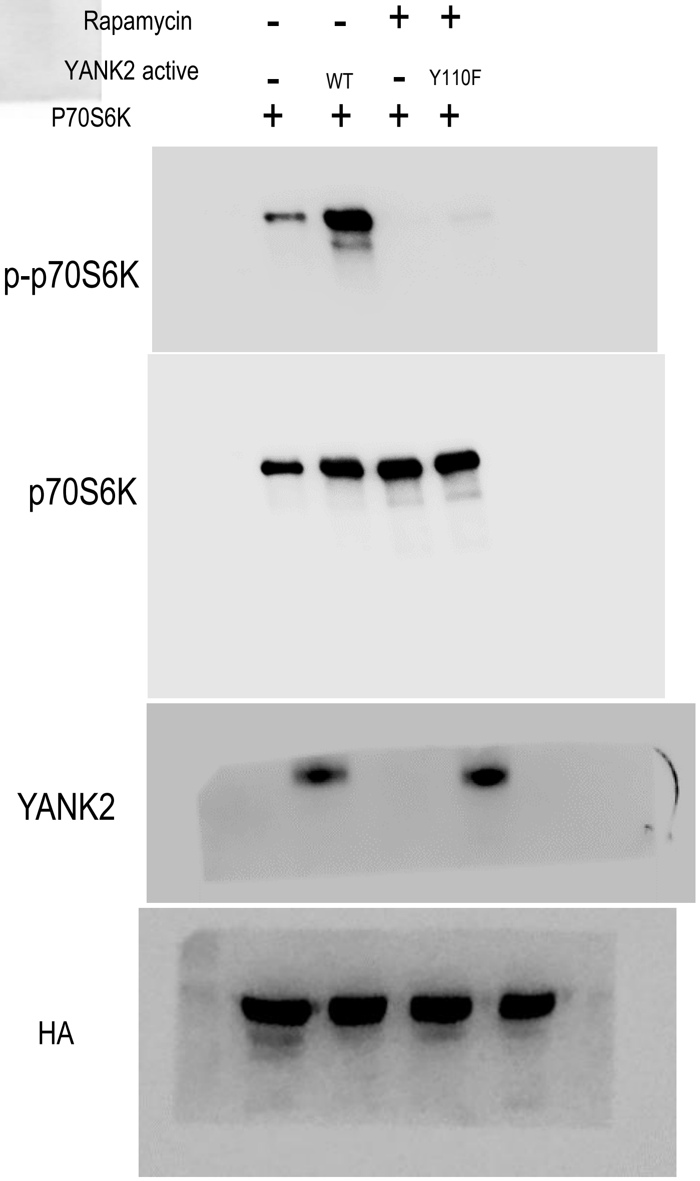

S2C. Same PVDF membrane

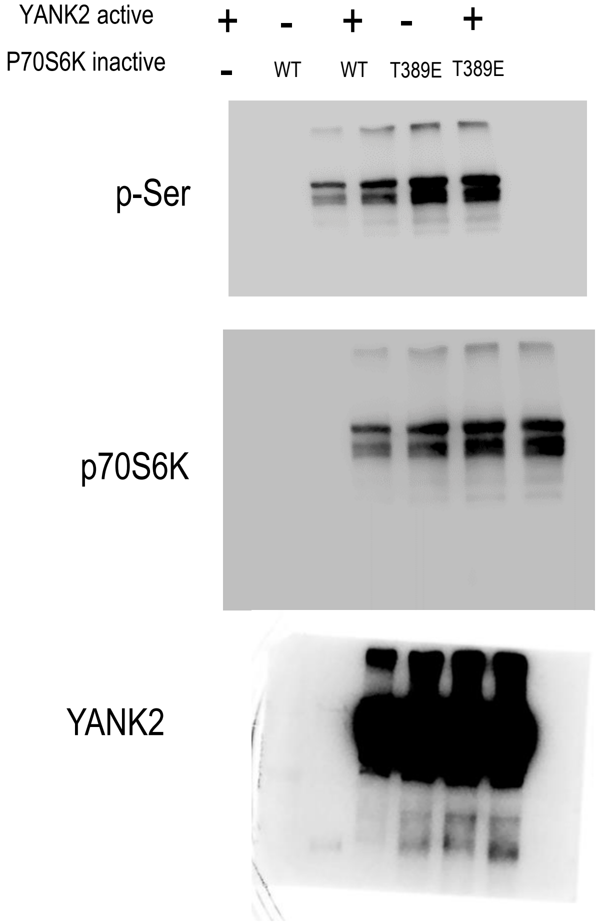

Figure 6 D.

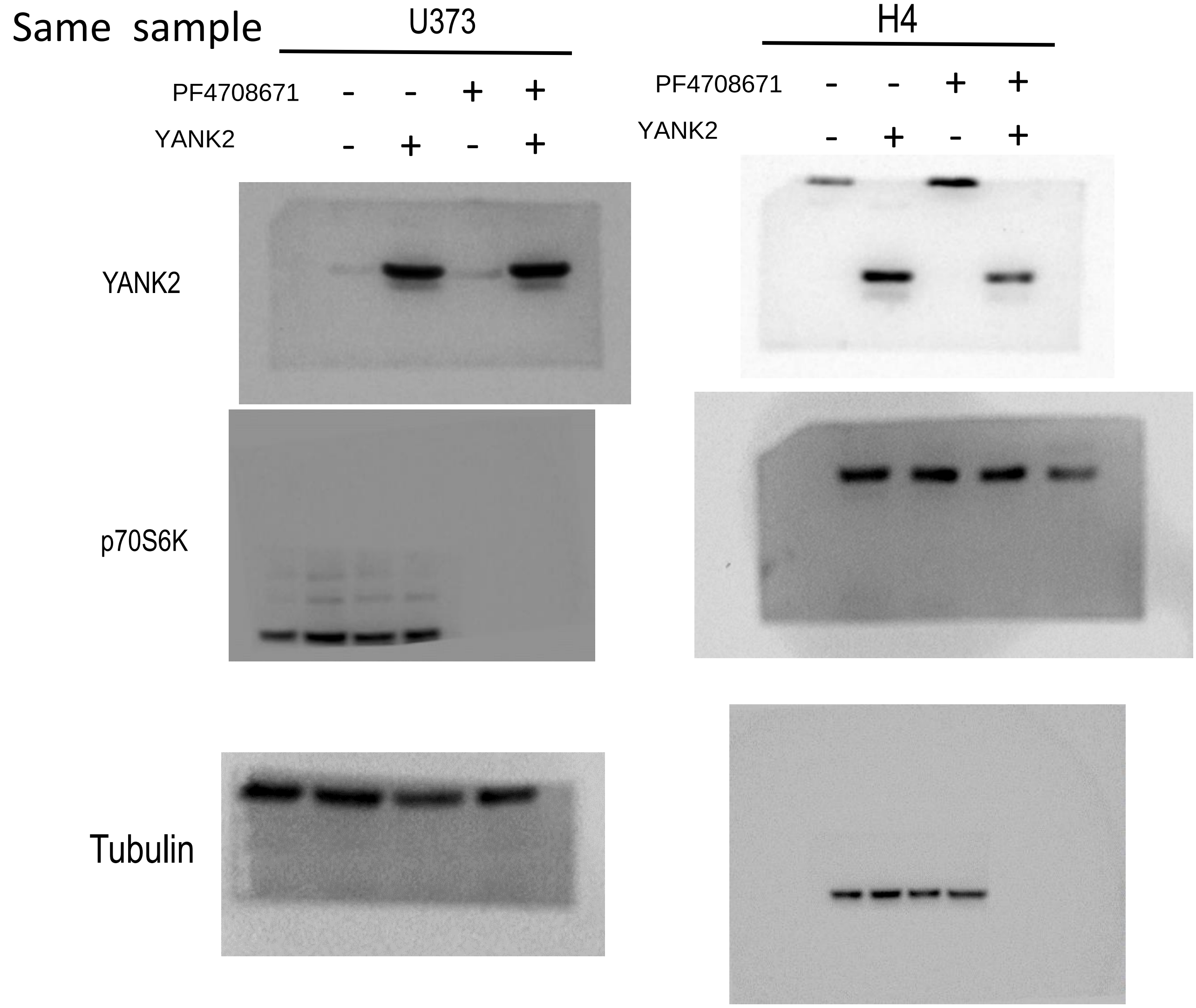
Same sample
U373
H4
PF4708671 - - + +
YANK2 - + - +
PF4708671 - - + +
YANK2 - + - +
YANK2
p70S6K
Tubulin

Figure 6 G Same sample

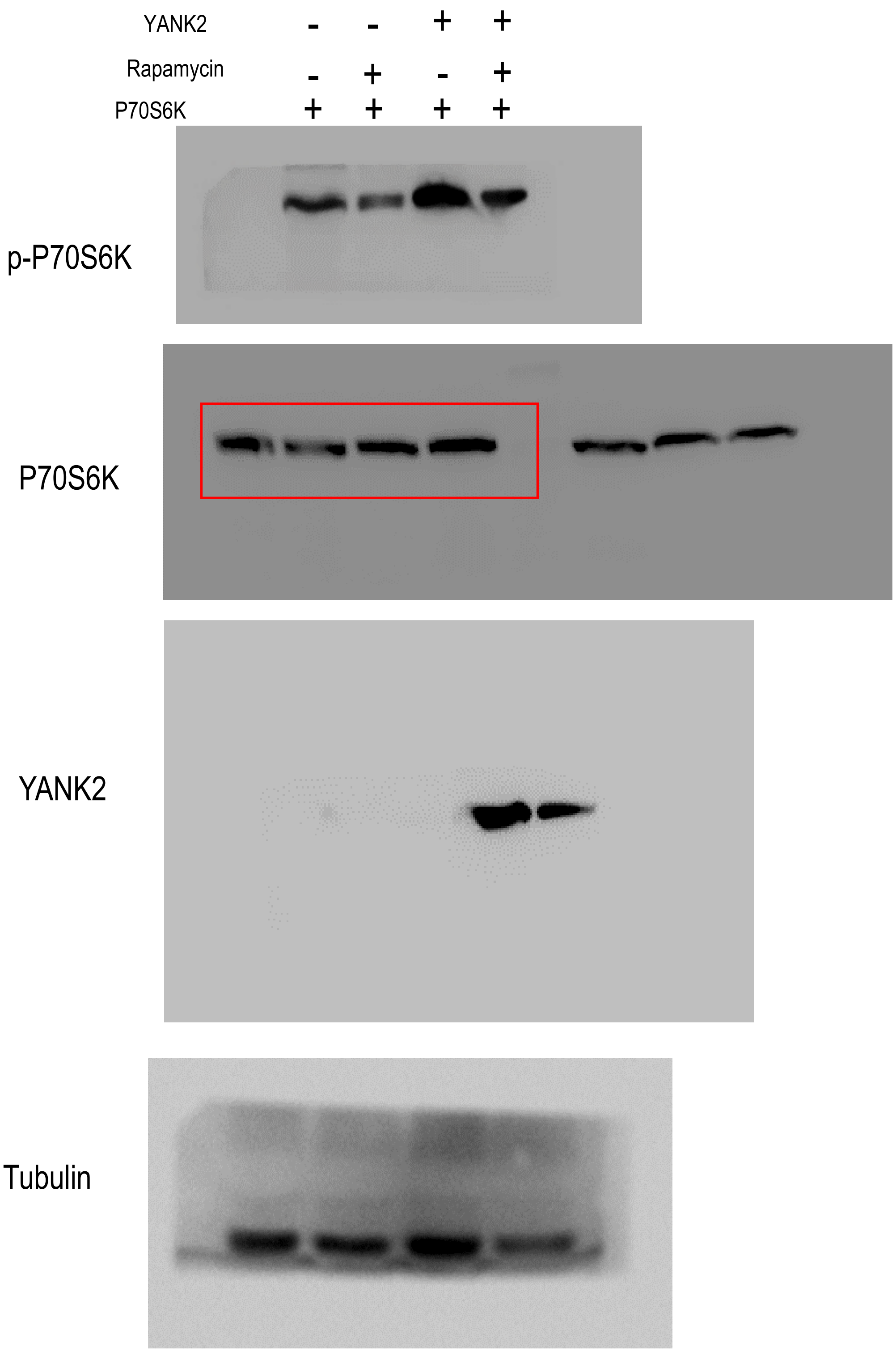

Supplemental Figure 1C.

Same PVDF membrane

YANK2

Tubulin

YANK2

Tubulin

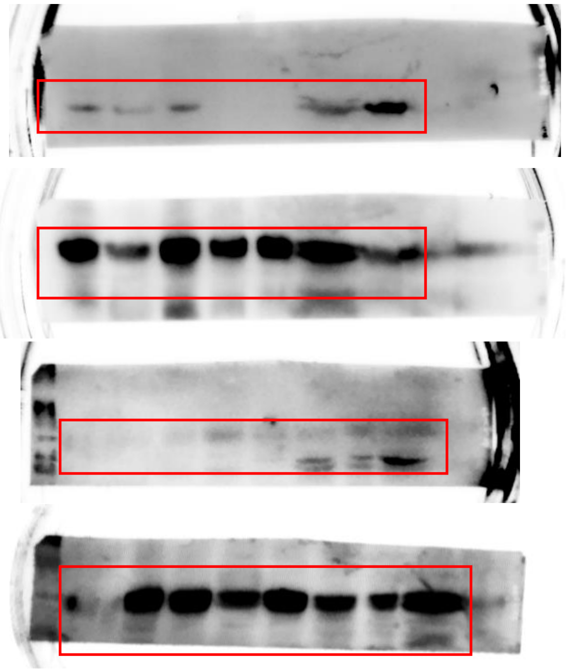

Supplemental Figure 1D.

Same sample

YANK2

Tubulin

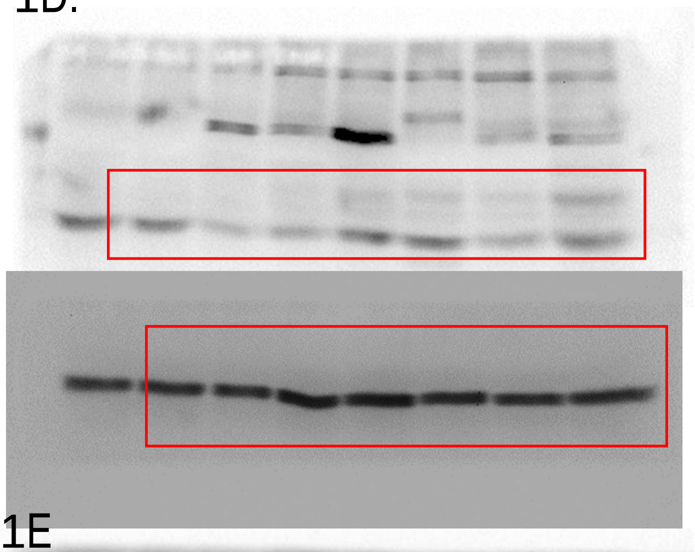

Supplemental Figure 1E

Same sample

Fyn

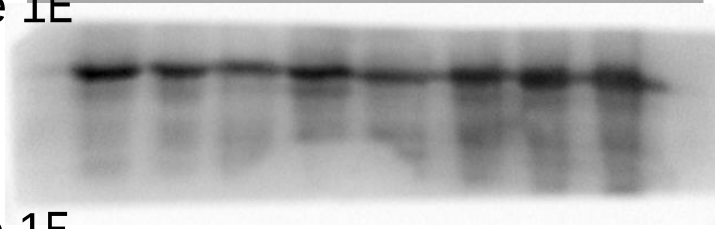

Supplemental Figure 1F

Same sample

p- P70S6K

Tubulin

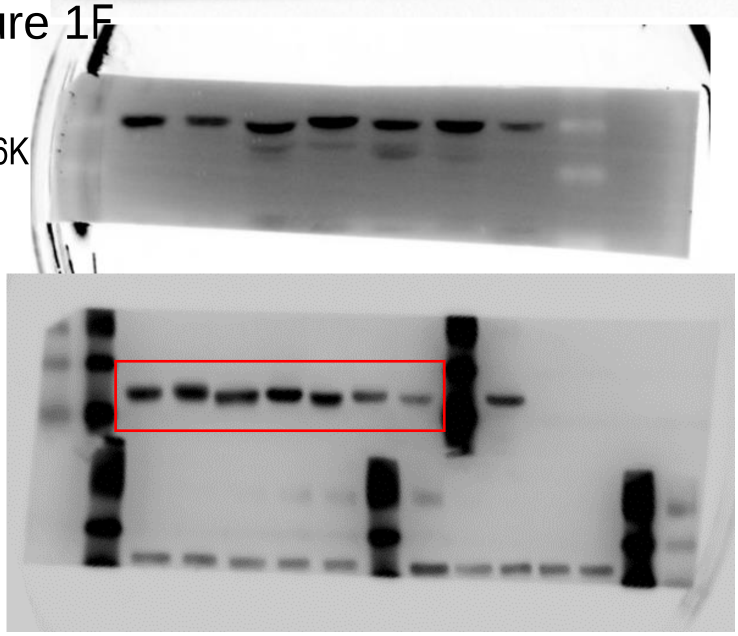

Supplemental Figure 2A.

Same sample

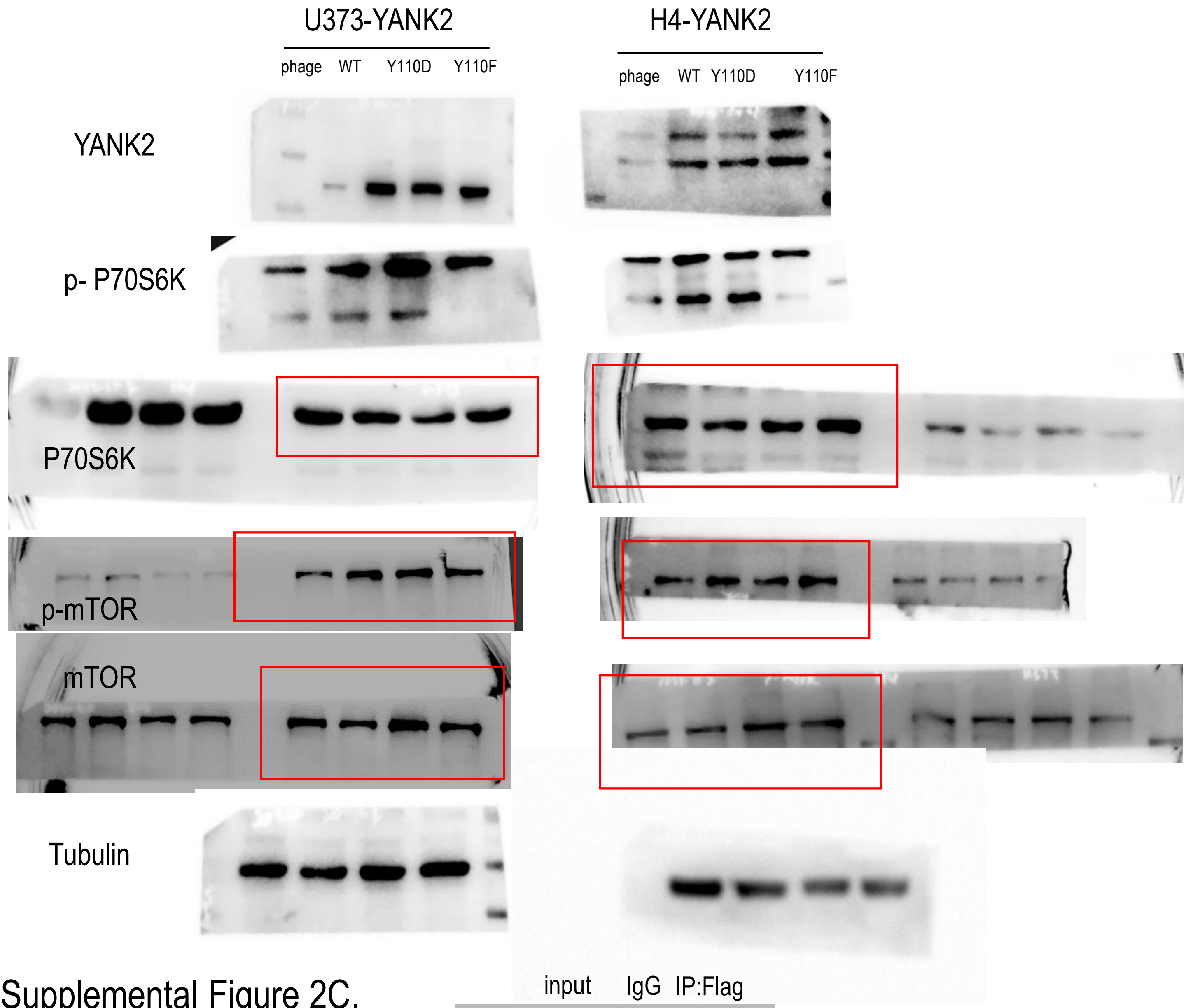

Supplemental Figure 2C.

Same PVDF membrane

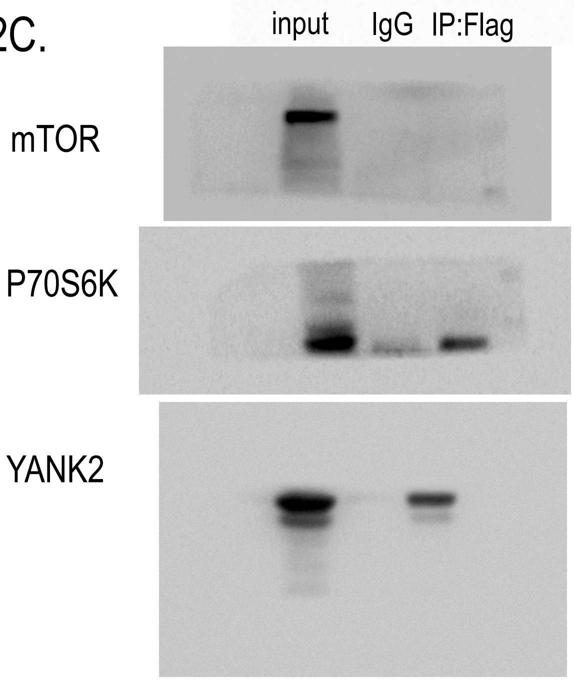

Supplement: Supplementary file 1 — Supplementary Figures. [file 41598_2024_61157_MOESM1_ESM.pdf]
