# Supplementary material for: YANK2 activated by Fyn promotes glioma tumorigenesis via the mTOR-independent p70S6K activation pathway
Source: Sci Rep. 2024 May 7;14:10507. doi: 10.1038/s41598-024-61157-5 (PMC11076283; doi:10.1038/s41598-024-61157-5)
Supplement: Supplementary file 2 — Supplementary Information. [file 41598_2024_61157_MOESM2_ESM.pdf]

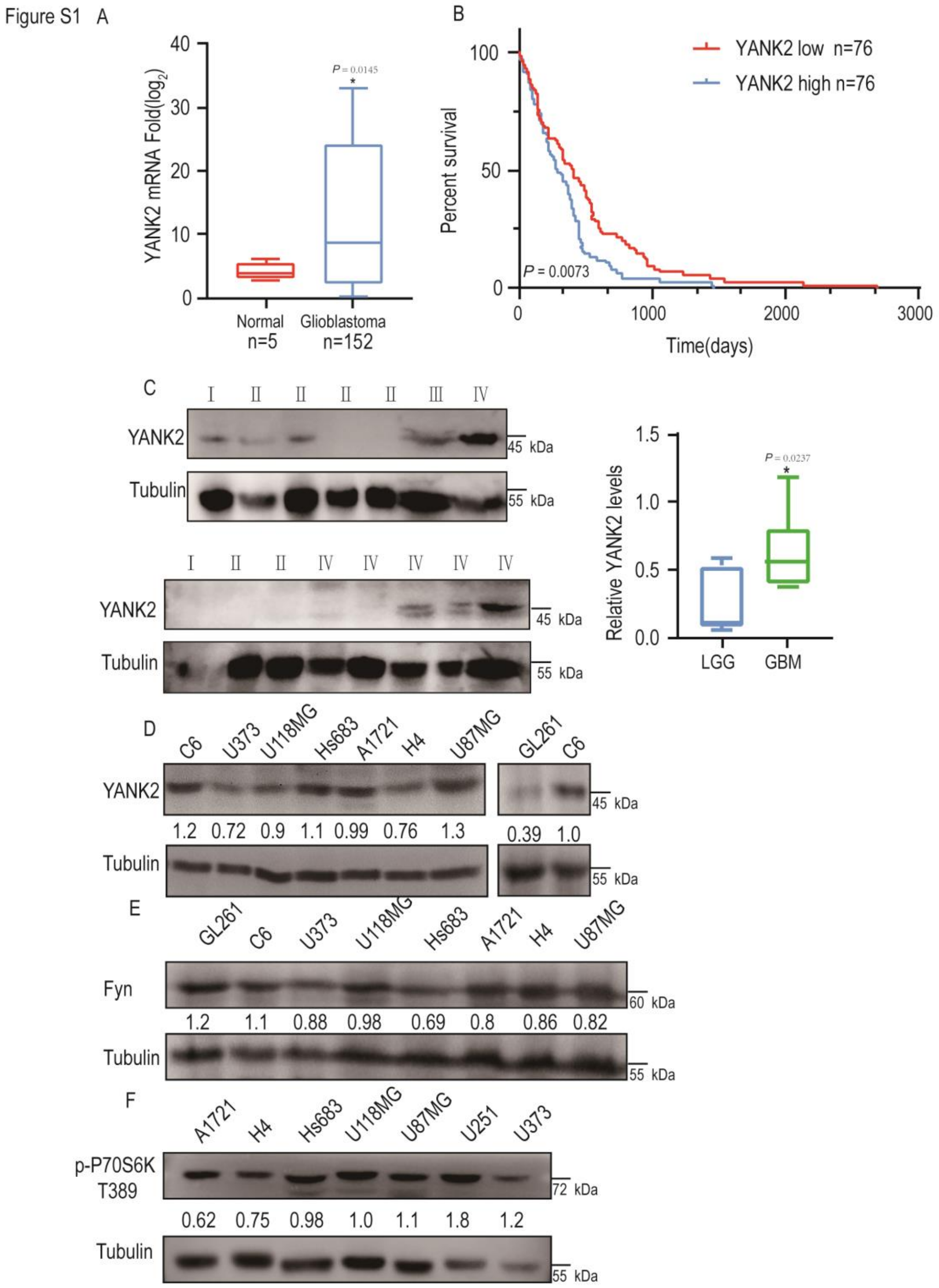

Figure S1 **High Expression of YANK2 Correlates with Tumorigenicity and Prognosis of GBM**

(A) YANK2 mRNA expression in GBM was higher than that in normal brain tissue (data comes from TCGA dataset);

(B) Kaplan-Meier survival analyses for YANK2 mRNA expression in GBM (data comes from TCGA dataset)

(C) Expression of YANK2 in 15 clinical glioma tissues were detected by WB;

(D) Expression of YANK2 were shown in different glioma cell lines cells by WB;
(E Expression of Fyn were shown in different glioma cell lines cells by WB;
(F) Expression of P70S6K were shown in different glioma cell lines cells by WB.

Figure S2

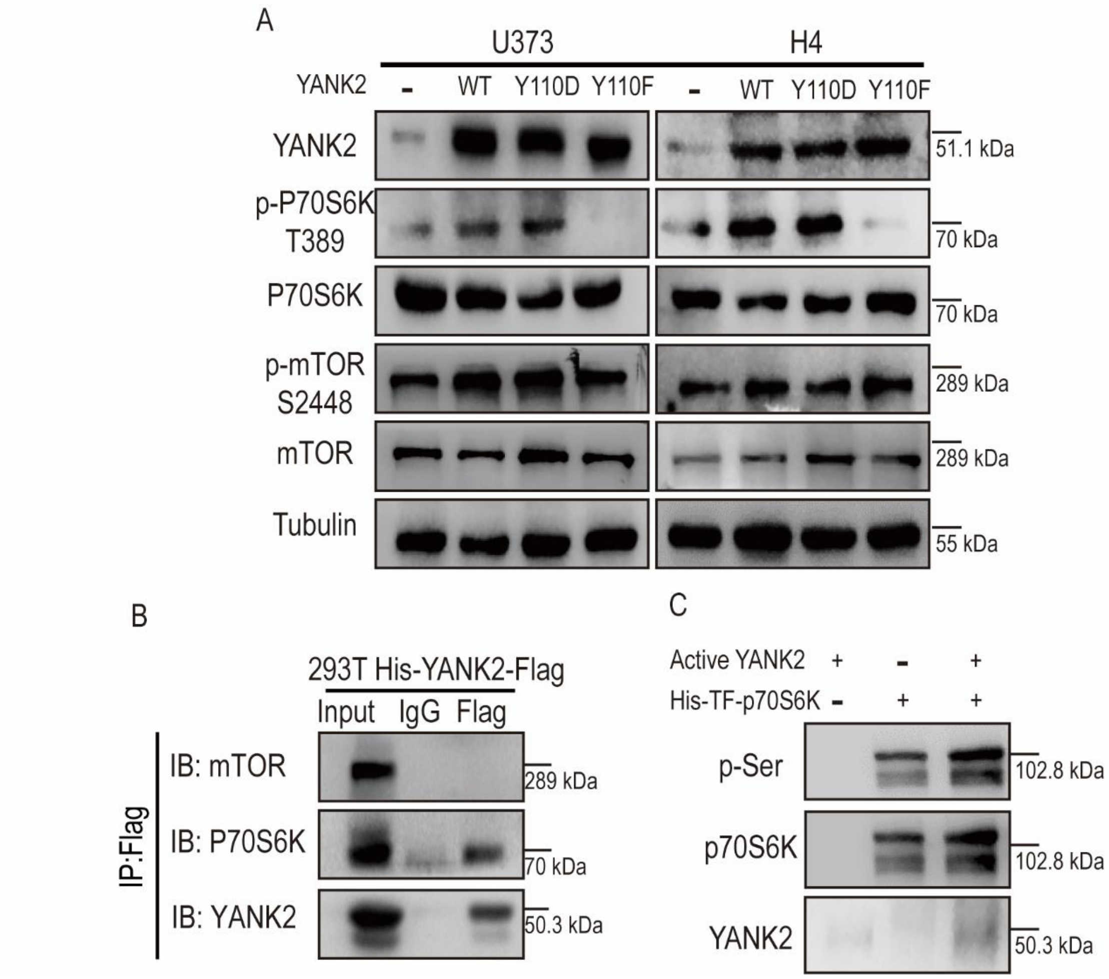

Figure S2 **YANK2 functions via p70S6K activation pathway independently of mTOR**

(A)Key molecules of mTOR signaling pathways in U373- or H4-YANK2 (WT, Y110F, Y110D) stable cells were detected by WB;

(B) pCMV-His-YANK2-Flag plasmid was transfected into HEK293T for 48 hrs, pulldown with anti-Flag and then probed with anti-mTOR and anti-P70S6K antibody;

(C) YANK2 phosphorylates p70S6K *in vitro*. Active YANK2 was obtained by IP with anti-Flag from HEK293T cells which were transiently transfected with pCMV-His-YANK2-Flag for 48 hrs and stimulated with EGF (80 ng/ml, 30 min), and then a kinase assay was performed with His-TF-p70S6K purified from bacteria as substrate, and phosphorylation of p70S6K was detected using anti-p-Ser antibody.

Figure S3

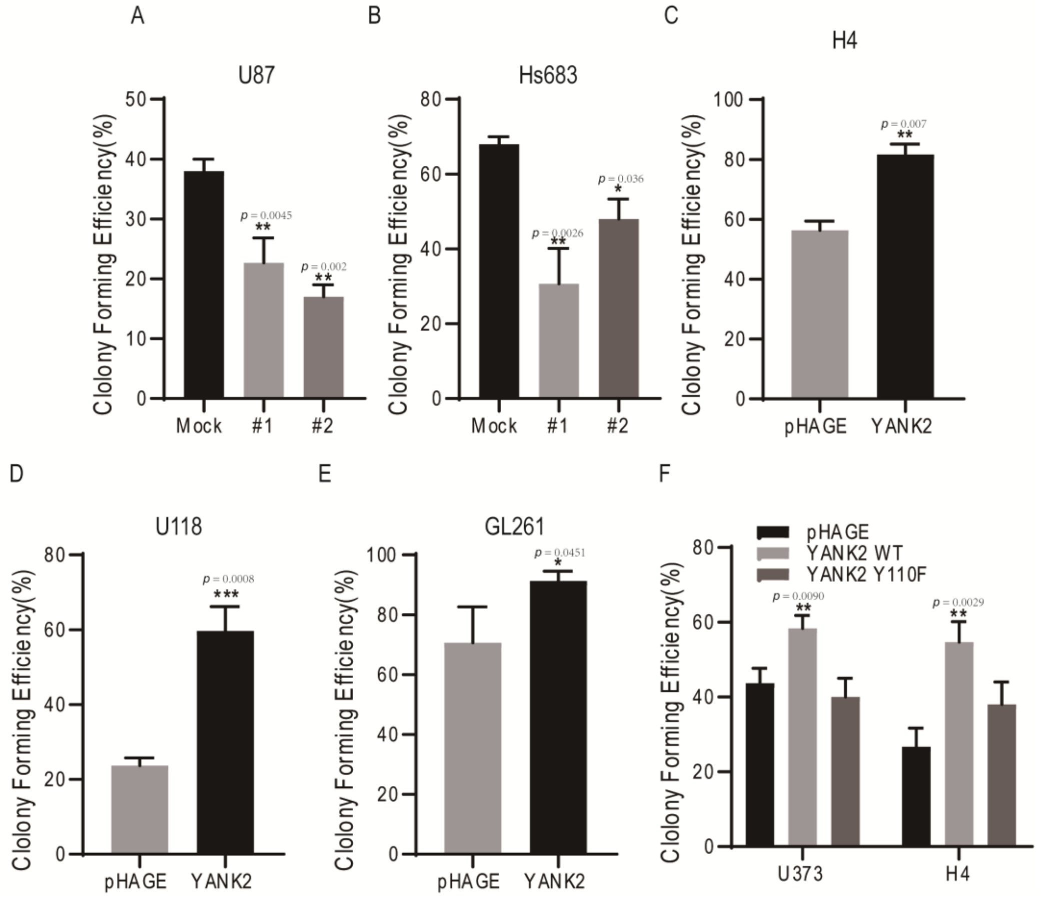

Figure S3 **Significance analysis of plate clones**

(A, B) The statistical analysis on the plate cloning results of Figure2B;

(C, D) The statistical analysis on the plate cloning results of Figure2D;

(E) The statistical analysis on the plate cloning results of Figure2G;

(F) Expression of p-P70S6K, YANK2 and Fyn were shown in U118MG sgFyn cells by WB.

Table S1. Oligonucleotide sequences

Primers	Sequence (5'→3')
YANK2-Y110D-F	GGAGGCGACCTGCGCGACCATCTGCAGCAGAATG
YANK2-Y110D-R	CATTCTGCTGCAGATGGTCGCGCAGGTCGCCTCC
YANK2-Y110F-F	GGAGGCGACCTGCGCTTCCATCTGCAGCAGAATG
YANK2-Y110F-R	CATTCTGCTGCAGATGGAAGCGCAGGTCGCCTCC
Fyn-K299M-F	GAAACACAAAAGTAGCCATAATGACTCTTAAACCAGGCACAATG
Fyn-K299M-R	CATTGTGCCTGGTTTAAGAGTCATTATGGCTACTTTTGTGTTTC
Fyn-F	CGCGGATCCATGGGCTGTGTGCAATG
Fyn-Y531F-R	CCGCTCGAGGTCGACCAGGTTTTCACCAGGTTGAAACTGGGGCTCTGTCGCGG
Fyn-R	AGACGTCGACCAGGTTTTCACCAGGTTG
p70S6K-F BamH1	CGCGGATCCATGTACCCATACGATGTTC
p70S6K-R Xbal	GCTCTAGATAGATTCATACGCAGGTGCT

shRNA sequences	Sequence (5'→3')
shYANK2#1	CAGAAGCGAGACACTAAGAAA
shYANK2#3	GCAGCAGAATGTGCATTTCAC
sgFyn#1	CACCGGGGACCTTGCGTACGAG
sgFyn#2	CACCGGGCTCCAGTTGACTCTATCC

Table S2 Antibodies

Antibodies	SOURCE	Catalogue
Anti-Alpha Tubulin Polyclonal antibody	Proteintech	11224-1-AP
Anti-FYN Monoclonal antibody	Proteintech	66606-1-Ig
Anti-p70(S6K) Polyclonal antibody	Proteintech	14485-1-AP
HRP-conjugated anti-mouse antibody	Proteintech	SA00001-9
HRP-conjugated anti-Rabbit antibody	Proteintech	SA00001-8
Anti-MYC tag Polyclonal antibody	Proteintech	16286-1-AP
Anti-MYC tag Monoclonal antibody	Proteintech	60003-2-Ig
Anti-Flag Monoclonal M2 Antibody	Merck	F1804
Anti-Flag Antibody	Merck	F7425
Anti-Phosphothreonine Antibody	Merck	AB1607
Anti-Phosphoserine Antibody	Santa cruz	sc-81514
Anti-YANK2 antibody	Abcam	ab154657
Anti-Phosphotyrosine Antibody	Cell Signaling	# 9381S

Anti-Phosphop70S6Kinase (Thr389) Antibody	Cell Signaling	#9234
Anti-Phospho-mTOR (Ser2448) Antibody	Cell Signaling	#5536
Anti-mTOR Antibody	Cell Signaling	#2972
Anti-p-ERK(T202/Y204) Antibody	Cell Signaling	#4370
Anti-ERK Antibody	Cell Signaling	#9102
Anti HA-Tag Antibody	Abclonal	AE008

Table S3. Phosphorylation Site identified by mass spectrometry

Proteins	Positions	Number of STY	Amino acid	Intensity	Ratio mod/base
MYP2_HUMAN	44	1	S	304480	NaN
TDT_HUMAN	219	1	S	160810	NaN
KALM_HUMAN	43	1	S	211540	NaN
PTPRG_HUMAN	45	1	S	19111	NaN
RPB2_HUMAN	1061	1	S	2528500	NaN
PRD15_HUMAN	915	2	S	2892600	NaN
ISCA2_HUMAN	29	2	S	609200	NaN
ASH1L_HUMAN	1003	2	S	217680	NaN
ASH1L_HUMAN	1008		S	0	NaN
ASH1L_HUMAN	1009		S	0	NaN
ADA30_HUMAN	3	1	S	7995000	NaN
SPTA1_HUMAN	2214	1	T	5191500	NaN
MYP2_HUMAN	40	1	T	1662500	NaN
PRD15_HUMAN	923	2	T	2892600	NaN
F214A_HUMAN	145	1	T	139480	NaN
IKIP_HUMAN	131	1	T	887960	NaN
ISCA2_HUMAN	27	2	T	609200	NaN
SLAI1_HUMAN	272	1	T	766840	NaN
FYN_HUMAN	420	1	Y	125640	0.55121
KLC1_HUMAN	10	1	Y	546920	NaN
DCPS_HUMAN	256	1	Y	1648600	NaN
DEFI6_HUMAN	483	1	Y	812170	NaN
PPIP2_HUMAN	52	1	Y	156990	NaN
ST32B_HUMAN	110	1	Y	57674	0.0027625
